# Essential Medicinal Chemistry of Essential Medicines

**DOI:** 10.1021/acs.jmedchem.0c00415

**Published:** 2020-04-30

**Authors:** Marta Serafini, Sarah Cargnin, Alberto Massarotti, Tracey Pirali, Armando A. Genazzani

**Affiliations:** Department of Pharmaceutical Sciences, Università del Piemonte Orientale, Largo Donegani 2, 28100 Novara, Italy

## Abstract

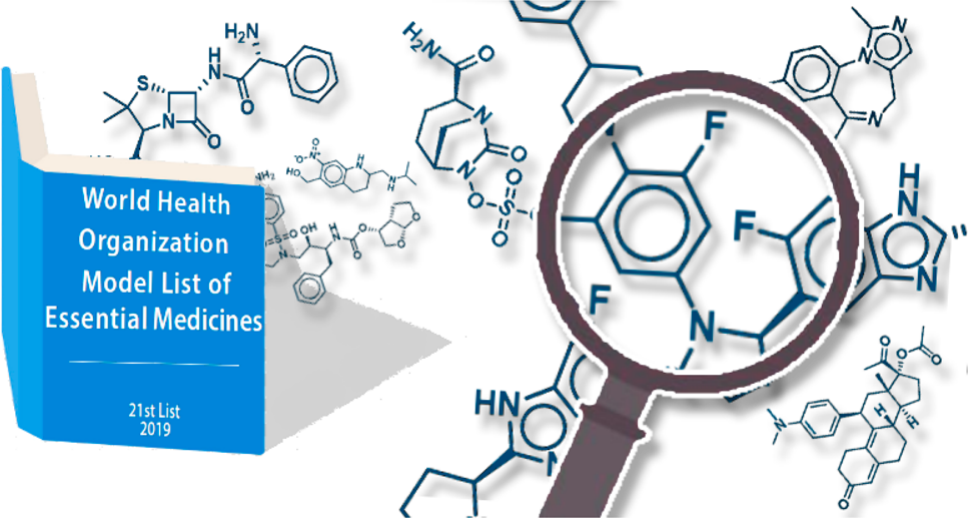

Since 1977, the World Health Organization publishes a list of essential medicines, i.e., those that satisfy the priority health care needs of the population and are selected with regard to disease prevalence and public health relevance, evidence of clinical efficacy, and safety, as well as comparative costs and cost-effectiveness. The Essential Medicines List (EML) is an invaluable tool for all countries to select those medicines that have an excellent risk/benefit ratio and that are reputed to be of pivotal importance to health. In the present perspective, we describe the chemical composition and the main features of the small molecules that are included in the EML, spanning from their origin, to their stereochemistry and measure of drug-likeness. Most and foremost, we wish to disseminate the importance of the EML, which can be both a helpful teaching tool in an ever-expanding world of medicines and an inspiration for those involved in pharmaceutical R&D.

## Introduction

Not all medicines are equal. They obviously differ in their structure, in their mechanism of action, and in their formulation. Most of all, though, medicines differ in the absolute clinical benefit they provide, which is in part but not solely dependent on the severity of the disease they target. While the absolute clinical benefit of a drug is important, it must be recognized that many diseases have a number of pharmacological treatments available. In this case, drugs can be differentiated by their added clinical benefit compared with the other alternatives. Drugs also differ in the quality and quantity of data that back up their health claims. Indeed, the efficacy and safety of a drug are supported by clinical evidence, which may be stronger or weaker. When the evidence is more robust, the drug is better, as lower is the uncertainty of what to expect from it. This latter concept is rapidly being adopted by decision-makers^1^ and is not dissimilar from the concept of evidence-based medicine developed to make decisions on single patients in the 1990s.^2^ Lastly, drugs differ in their cost. The fact that drugs cost differently, that diseases are different in their severity, and that the different medicines have different efficacies leads to the concepts of cost-benefit and cost-effectiveness. In brief, these are indexes that show that a similar health gain (for example, a life-year gained in good health) may cost differently according to the drug being used or the disease being cured.

The above considerations, albeit simplified, are the key drivers to decision-making in the regulatory arena, when deciding whether to make available a particular medicine. They are even more important when making choices regarding drugs from a global health perspective, in a situation in which economic resources are often insufficient. This was recognized back in 1975 by the World Health Assembly, which asked the World Health Organization (WHO) to assist member states in choosing their medicines.^3^ The initial indication from the World Health Assembly was to help select and procure essential medicines, assuring good quality and reasonable cost.^3^ Furthermore, as mentioned in the speech of the WHO Director General at the time, the differentiation of essential and inessential medicines would also have “stimulated research and development to produce new drugs adapted to the real health requirements of developing countries”.^4^ Essential medicines (EMs) are nowadays defined as those that satisfy the priority health care needs of the population and are selected with regard to disease prevalence and public health relevance, evidence of clinical efficacy and safety, as well as comparative costs and cost-effectiveness.^5^

In the 1970s, no more than a dozen countries in the world had what would be considered nowadays as medicines formulary,^6^ which is a list of drugs that can be prescribed and are reputed essential for that particular country. This lack of global awareness that not all medicines were essential brought WHO to compile the first list of 205 items (186 medicines), published in 1977, which became known as the st (EML). Since then, the scope of the EML has slightly drifted from its original objectives, making it even more relevant for global health as a tool to reduce the gap in availability, affordability, and access to medicines. The list has now been updated 21 times, usually on a biannual basis, with the most recent list issued in 2019 that includes 459 items.^7^ While the general definition of EMs is the driver when deciding when a medicine should be included in the list, medicines are so different among them that it is not only cheap drugs with an excellent risk/benefit profile used for high prevalence diseases that are listed. For example, imatinib, given its magnitude of benefit in chronic myeloid leukemia (CML), is present on the list despite the fact that CML is a rare hematological disorder. Furthermore, trastuzumab, rituximab, and new cancer immunotherapies for melanoma, which are, in principle unaffordable, in most areas of the world, are included due to their efficacy. Importantly, inclusion on the list, it has been advocated, improves access, and affordability. The list also includes drugs for which specialized care is necessary (grouped in a complementary list; for example, those drugs that require a specialist or special monitoring) and those that are essential for children (children’s list). The list can be used by single entities (e.g., nations, nongovernative organizations, etc.) to create a more targeted formulary, determined by local needs and resources.

It is reported that, nowadays, four countries out of five have National Essential Medicines Lists.^6^ Countries may choose to adopt the full EML, to choose only some drugs from the list, or to implement the list to provide further healthcare, depending on strategic choices and/or resources.

One of the characteristics of the EML is to include only a single drug if there are several alternatives, for example, me-too drugs, that show similar clinical performance.^8^ On the actual list, the presence of equivalent alternatives on the market is indicated by an open square box next to the name of the listed drug. As an example, only omeprazole is present on the list, but a square box next to its name signifies that all other proton pump inhibitors (e.g., pantoprazole, rabeprazole, esomeprazole) with an identical fourth level ATC (anatomical therapeutic chemical classification system) code show similar performance and may be used as alternatives. The choice of which medicine to list with a square box is driven by the availability of the best evidence for effectiveness and safety, by priority dates on the market and/or by the notion that some drugs will most likely be cheaper worldwide. This strategy avoids having a very long list and reduces the risk of investing resources in duplicate medicines. Furthermore, it may be a tool in some geographical areas to optimize procurement. The square box is used sparingly with antibiotics.

Inclusion on the list is a rather straightforward process. An applicant (which can be represented by any person or organization, including pharmaceutical industries) may apply for the inclusion of a particular medicine or a class of medicines by providing the data that support their essentiality.^9^ It is preferable that these applications include systematic reviews of all available data and meta-analyses that show the absolute or comparative magnitude of benefit. An Expert Committee, chosen based on equitable geographical representation, gender balance, and professional competences in order to provide different perspectives, is then summoned to decide on the applications which are elaborated by an *ad hoc* secretariat.^10^ In 2019 the Expert Committee considered 65 applications and recommended the addition of 28 new medicines on the EML, 23 new medicines on the children’s list, 16 new formulations, and 25 additional indications for drugs that were already listed. Not all applications are accepted, and 21 were rejected in 2019. Furthermore, medicines can be deleted from the list, and 9 were deleted in 2019. Requests by applicants and decisions are all available on the WHO Web site.^9^ The actual list is a book, freely downloadable from the WHO site, composed of chapters that deal with specific pharmacological classes or diseases.^8^ In each chapter, the drugs are listed together with their intended use, strength, and formulation. The list ends with an index that contains all items in alphabetical order. Moreover, on February 27th, 2020, an electronic version of the list was launched in a beta phase.^11^

Previous reviews have concentrated on one or more aspects concerning the medicinal chemistry of marketed drugs.^12^ Yet, again, not all medicines are equal. Therefore, in the present perspective, we decided to evaluate the medicinal chemistry characteristics of the drugs included in 21st WHO EML.^8^

### Overview of the Items on the List

**Figure 1:**
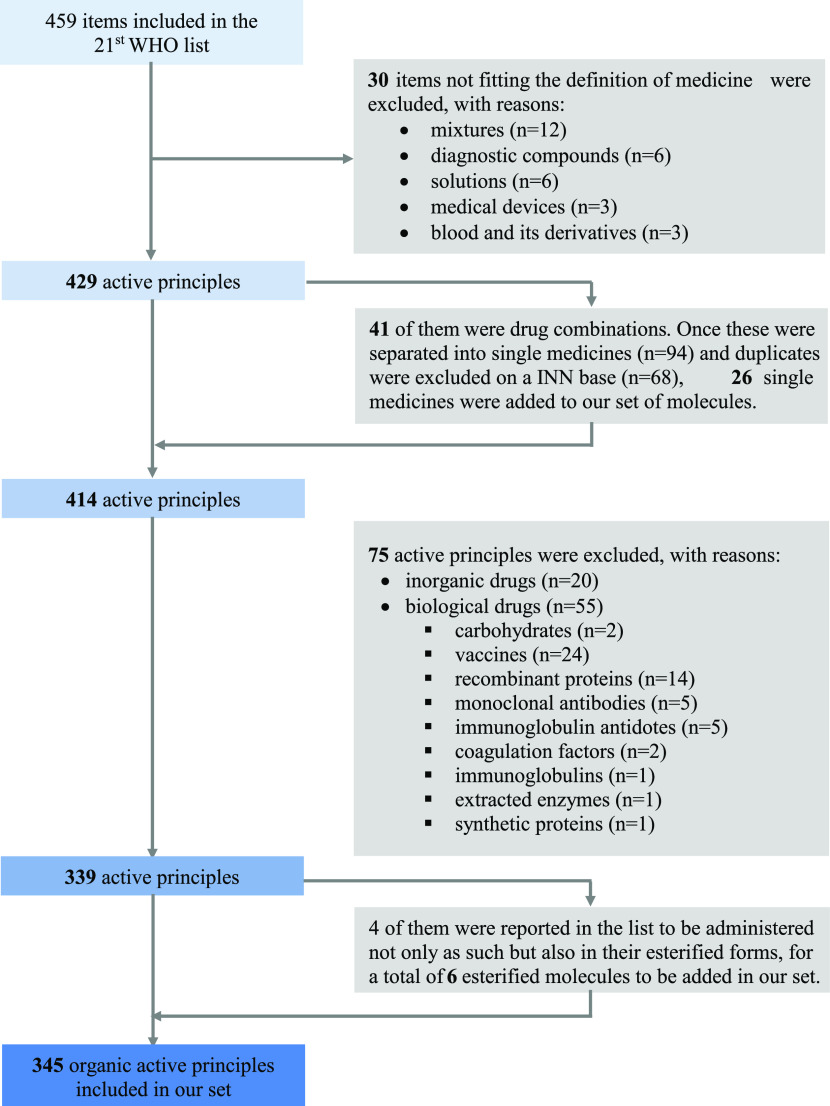
Flowchart depicting the items on the EML and their exclusion to reach a unique, organic chemistry subset.

The index of the 21st WHO Model List of Essential Medicines includes 459 items (Figure 1), and not all these fit the definition of medicine. For example, a number of diagnostic agents are present (e.g., fluorescein, amidotrizoate, iohexol, meglumine iotroxate, barium sulfate, tuberculin), as well as some medical devices (e.g., condoms, diaphragms, copper-containing devices). While the presence of condoms and diaphragms is surprising in a list intended for medicines, it is likely that such inclusion was based on the crucial importance that sexually transmitted diseases and family planning have on global health. The list also contains blood derivatives and solutions (e.g., water for injection or oral rehydration salts). Given that we intended to perform an analysis of the medicinal chemistry of drugs, the above items were obviously excluded from any analyses. Such exclusion was also extended to a few mixtures, which are difficult to characterize from a chemical viewpoint (e.g., senna, chlorine base compound).

This left 429 items, including 41 products, which are drug combinations (e.g., ombitasvir + paritaprevir + ritonavir). To explore the medicinal chemistry of EMs, we decided to analyze the single molecules in the combinations separately. The exclusion of duplicates (that are molecules present alone and in combination or in more than one combination) yielded 414 unique active principles.

Given the focus of the review, we then decided to exclude all biological entities. The list includes 55 biological entities (13% of all chemical and biological entities). The most represented entities in the protein group are vaccines (*n* = 24), recombinant proteins (*n* = 14), monoclonal antibodies (*n* = 5), immunoglobulin antidotes (*n* = 5), and carbohydrates (*n* = 2; enoxaparin and heparin sodium). It is likely that, during the years, the cost of production somehow influenced the addition of recombinant proteins and monoclonal antibodies to the EML as the percentage of these products on the market is significantly higher. Alongside production costs, it is possible that the overall cost of biological medicines on the market has also slowed the uptake of such agents on the list. Biological and advanced therapies approved by the European Medicines Agency (EMA) between 2018 and 2020 account for 36% of all new entities,^13^ strongly suggesting that these drugs are underrepresented in the EML. Such under-representation may also be a consequence of the slow uptake in the EML of novel technologies due to the need to gather sufficient evidence.

Twenty drugs out of the 414 chemical entities (about 5%) are represented by inorganic drugs (Table 1). While some of these compounds are for rehydration, it still appears to be a high number compared with the general perception of medicines.

**Table 1:** Inorganic EMs

inorganic active principle	WHO EML section/s
arsenic trioxide	cytotoxic medicines
cisplatin	
calcium salts	vitamins and minerals
iodine	
sodium fluoride	
ferrous salt	antianemia medicines
lithium carbonate	medicines used in bipolar disorders
nitrous oxide	general anesthetics and oxygen
oxygen	
magnesium sulfate	anticonvulsants/antiepileptics
potassium chloride	solutions correcting water, electrolyte, and acid–base disturbances
sodium chloride	
sodium hydrogen carbonate	
potassium ferric hexacyano-ferrate(II)	antidotes and other substances used in poisonings
sodium nitrite	
potassium iodide	antifungal medicines; thyroid hormones and antithyroid medicines
potassium permanganate	dermatological medicines
selenium sulfide	
sodium thiosulfate	dermatological medicines; antidotes and other substances used in poisonings
zinc sulfate	medicines used in diarrhea

We then looked at each drug in the main text of the EML to establish its salified form. Briefly, 116 organic drugs are inserted in at least one salified form, while 8 drugs contain ammonium quaternary salts. Twenty different salts are represented on the list, and the most abundant one is chloride (*n* = 45), followed by sodium (*n* = 29) and sulfate (*n* = 14). Surprisingly, only traditional inorganic cations are present, while no organic bases are listed, despite being nowadays preferred to avoid mineral load to patients.^14^ On the other hand, some organic carboxylic acids (e.g., maleate, lactate, citrate) are listed. Only one liposomal formulation is present on the list (that is amphotericin B sodium deoxycholate). A number of organic drugs are listed in different salified forms (e.g., amlodipine maleate, mesylate, and besylate) or as different ammonium quaternary salts (that are neostigmine bromide and methyl sulfate). These were considered as duplicates in the following analyses that were performed solely on the drug core.

Using the same approach, we realized that there was an incomplete correspondence between the index and the main text of the list when drugs presented a modified International Nonproprietary Name (INNM, that is a two-word name in which the first word indicates the active principle and the second word indicates the esterification,^15^ e.g., beclomethasone dipropionate), possibly as a result of errors in the index. We therefore decided if a drug presented two esterifications or was mentioned both as the core drug and as an INNM to include both. This occurred for 4 drugs and led to the addition of 6 entities, leading to 345 distinct organic drugs, which were the only molecules we concentrated on in our analyses (Figure 1).

The 22 INNMs included 10 different esters with the most abundant ones being acetate (*n* = 4), enanthate (*n* = 3), palmitate (*n* = 3), and succinate (*n* = 3). Moreover, a carbonate (tenofovir disoproxil) and a phosphate ester (dexamethasone phosphate) are also inserted. Finally, in one case (dabigatran etexilate), the INNM refers to both a carbamate moiety and the ethyl ester.^16^

#### Initial Description of Organic Drugs in the EML

**Figure 2:**
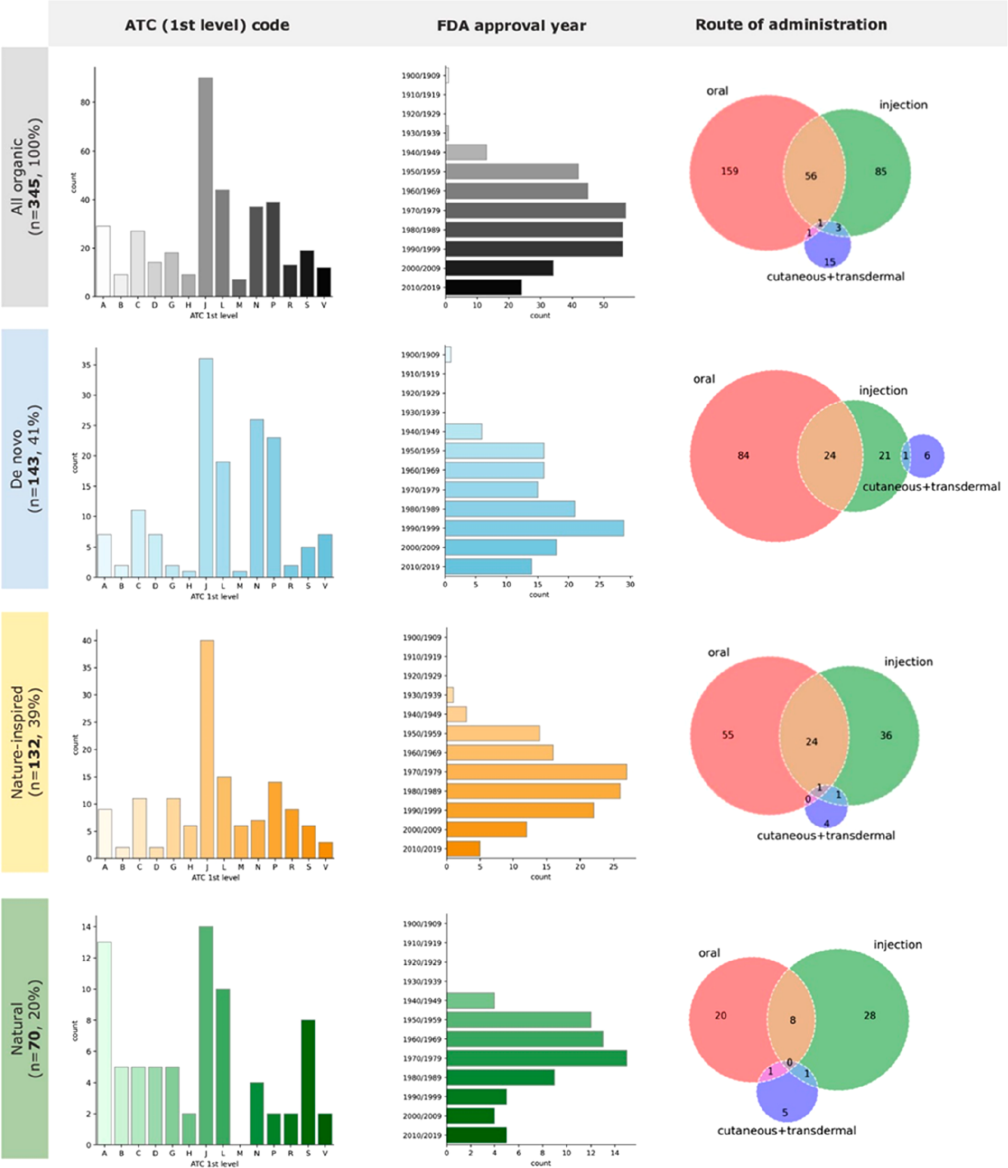
Classification of EML organic drugs according to their origin, ATC (1st level) codes, FDA approval year, and route of administration.

To understand the limits of our analysis, we first evaluated the therapeutic indications of the drugs in the EML. Figure 2 lists the frequency of drugs per the first-level ATC code (that is the anatomical district on which drugs act).^17^ As it can be seen, the most frequent first-level ATC codes are J (anti-infectives for systemic use, *n* = 92, 27%), L (antineoplastic and immunomodulatory agents, *n* = 44, 13%), P (antiparasitic drug, insecticides and repellants, *n* = 39, 11%), and N (nervous system, *n* = 37, 11%). Antibiotics and correct use of them represent a strong focus of the EML Expert Committee, and new tools to classify them for appropriateness are continuously updated.^18^ Given that J and P ATC codes are justifiably heavily represented in the EML, it is likely that the subdivision of ATC codes in the list does not mirror the approved drugs that are usually analyzed in other manuscripts (e.g., FDA-approved drugs or EMA-approved drugs). As an indirect comparison, we determined the ATC codes of drugs approved by EMA between 2018 and 2020^13^, and only 14% are in the J category and none in the P category, while the L category accounts for 26%. These strong differences in drug classes should be kept in mind when comparing our analyses to those reported by others on either more recent data sets or deriving from all approved drugs.

A second item that needs to be considered when reading the review is the first approval date of the drugs on the EML. Indeed, it usually takes a significant amount of time for a drug to collate a sufficient amount of evidence to convince the EML panel of its magnitude of benefit. The median year of approval from the Food and Drug Administration (FDA) of the drugs on the EML is 1981, with only 58 drugs (17%) approved after 2000 (Figure 2). In recent years, though, drugs that have shown the important magnitude of benefits such as antihepatitis C drugs (e.g., daclatasvir, sofosbuvir, glecaprevir) or cancer immunotherapies for melanoma (e.g., nivolumab, pembrolizumab) have rapidly reached the list.

Finally, from the main text of the list, all routes of administration of organic chemical entities were analyzed. Three subsets of EMs were created (oral: *n* = 217 medicines; injectables: *n* = 145; cutaneous: *n* = 20), as this allowed us to evaluate particular characteristics in subsequent analyses (for example, obedience to Lipinski’s rule of 5) (Figure 2). Fifty drugs were listed with routes of administration that did not fit in any of the above (e.g., rectal, ocular, vaginal). Obviously, a single agent could have been inserted in more than one list (e.g., sulfasalazine is listed in its oral and rectal formulation).

**Figure 3:**
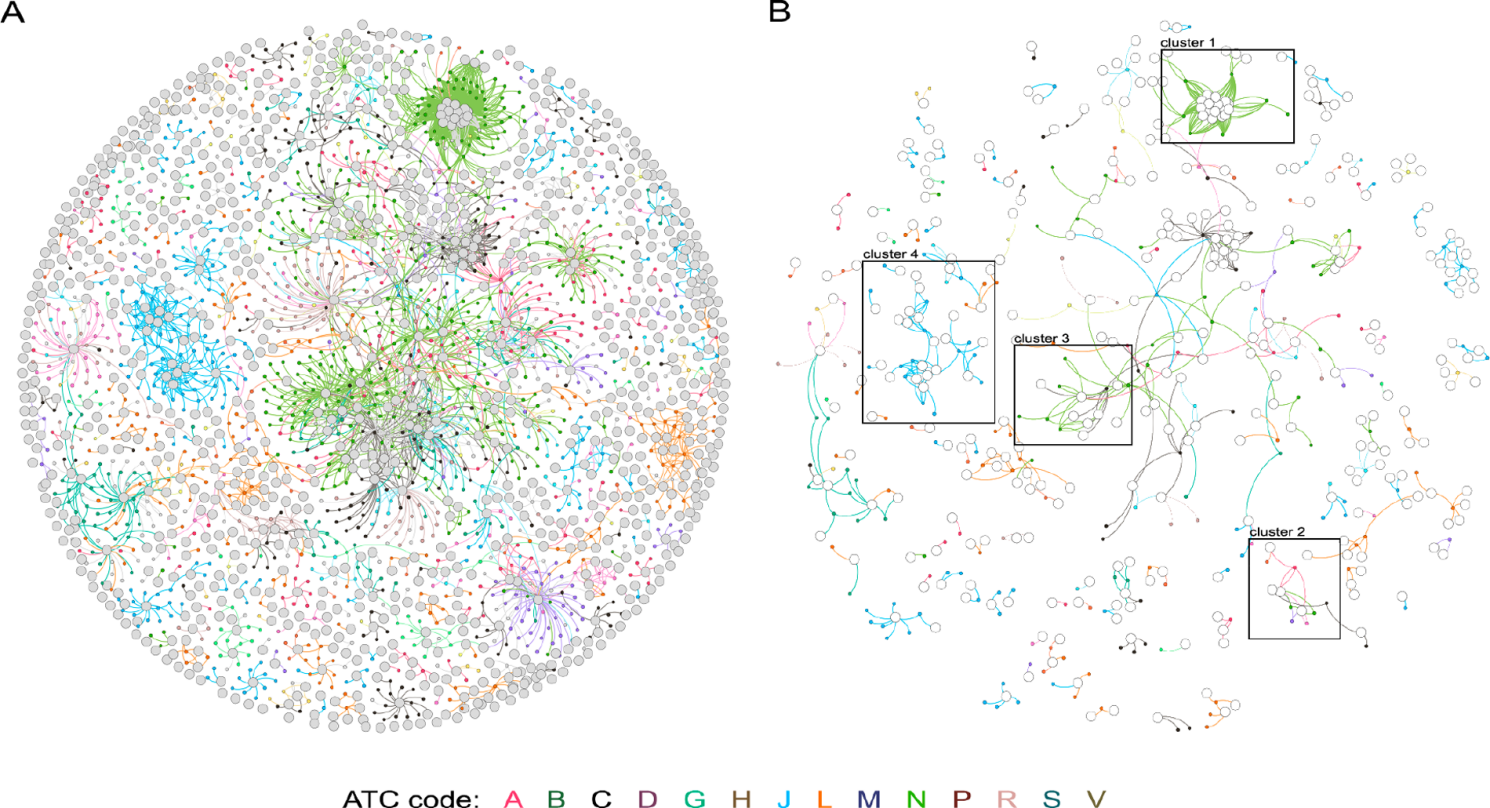
DT network of all approved drugs (A) and of EMs (B). The DT network is generated by using the known associations between drugs and targets extracted from the DrugBank database.^21^ As of March 2, 2020, DrugBank (version 5.1.5, released 2020-01-03) includes 2635 approved small molecule drugs and 1367 approved biologics. Additionally, 1148 nonredundant proteins (that are drug target/enzyme/transporter/carrier) sequences are linked to these drug entries. Small and big circles correspond to drugs and target proteins, respectively. A link is placed between a drug node and a target node if the protein is a known target of that drug. Drug nodes and connecting links are colored according to the ATC 1st level code of the drug. A bigger representation of the DT network is present in the Supporting Information.

We have also analyzed the qualitative drug-target (DT) network of the EML in comparison to one of the approved drugs for human use, in analogy to what was performed previously.^19^ Briefly, in Figure 3, big gray circles indicate biological targets, small circles indicate single drugs, and lines indicate DT interactions. Drug nodes and connecting links are colored according to the first-level ATC code. The left panel refers to all FDA-approved drugs, while the right panel indicates EMs. It is obvious that there is a strong reduction of both drugs and targets in the EML, given the lower density of big and small circles in the right panel. In part, this is given by the selection of drugs that show clinically similar performances and exclusion of drugs for disorders that the EML does not consider of strategic importance. Cluster 1, for example, is a good representation of this and is referred to as benzodiazepines and GABA-A receptor subunits. On the market, there are tens of benzodiazepines, used for epilepsy, anxiety, preoperative sedation, alcohol abstinence syndromes, and sleep disorders. In the EML, only 3 benzodiazepines are listed: lorazepam for epilepsy, diazepam for anxiety, epilepsy, and palliative care, and midazolam for preoperative sedation, epilepsy, and palliative care. In the same cluster, the list also includes one barbiturate (phenobarbital), halothane, isoflurane, and propofol for anesthesia, three drugs that are thought to act partially through the GABA receptor. A similar situation is represented by clusters 2 and 3 that refer to drugs that target cyclooxygenases and dopamine/serotonin receptors/transporters, respectively. Similarly, cluster 4 shows that only a proportion of cephalosporins and penicillins are selected for the list. We did not evaluate whether listed medicines were mainly discovered with a target-based approach or a phenotypic based approach, and refer to a recent review by Eder et al. that classified first-in-class FDA-approved drugs from 1999 to 2008 on this basis.^20^ The main reason hampering this analysis and not allowing to understand whether EMs are discovered mainly starting from the target or from the molecule is given by the fact that it is not always the first-in-class drug, which is listed in the EML.

#### How Many EMs Come from Natural Sources?

The number of drugs that are either found in nature or whose development was somehow influenced by natural substances is usually said to be high.^22^ We, therefore, evaluated the origin of EML drugs subdividing them into three categories: (i) natural (N), (ii) nature-inspired compounds (NI), and de novocompounds (DN). N compounds were defined as those molecules that can be found in nature and have not been further modified (e.g., all-*trans* retinoid acid, ascorbic acid, atropine, codeine, folic acid), while NI compounds were defined as those that derive from a natural source whose structure has been modified (e.g., rifampicin, simvastatin, vinorelbine) or compounds whose pharmacophore derives from a natural molecule or whose design has been presumably inspired by nature (e.g., levonorgestrel, bisoprolol, methadone). Last, DN molecules are those with no obvious relation to a natural source (e.g., lorazepam, lenalidomide, furosemide, fluoxetine, carboplatin). A list of the categorization is present in the Supporting Information.

As shown in Figure 2, de novo compounds account for 143 entities, the nature-inspired for 132 entities, and the natural ones for 70 compounds. This classification was performed solely on the chemical structure or on known derivation in its discovery and did not consider the methodology currently used for its production. For example, a drug derived from a natural source that is currently produced using a synthetic approach (e.g., ascorbic acid, salicylic acid) was inserted in the natural class. It should be noticed that the nature-inspired group contains a small subset of drugs in which substantial synthetic modifications have led to compounds that are only marginally related to the original compound (e.g., fentanyl, methadone, tropicamide). Procaine and other local anesthetics (lidocaine, bupivacaine, tetracaine) were classified in the de novo compounds, despite being perceived as simplified analogues of cocaine. Indeed, the structure of the latter was fully elucidated only in 1924, long after the discovery of procaine (1905), prompting us to consider local anesthetics as de novo drugs.^23^

A similar categorization was performed by Newman and coauthors^22a^, analyzing the new drugs approved by the FDA in the period 1981–2014. While the percentage of the de novo (49% in FDA-approved drugs vs 42% in EML) and nature-inspired (44% vs 38%) drugs in the two analyses are somehow comparable, natural compounds (7% vs 20%) differ significantly. Obviously, there is some arbitrariness in the classification, also given by the different background knowledge of the authors and by definition made to subdivide the drugs in natural, nature-inspired, and de novo compounds. This cannot, though, account for the 3-fold overrepresentation of the natural compounds in the EML compared with Newman and coauthors^22a^ and also because there is little arbitrariness in this category.

#### Composition of Organic EML Drugs

##### Elemental Composition

**Figure 4:**
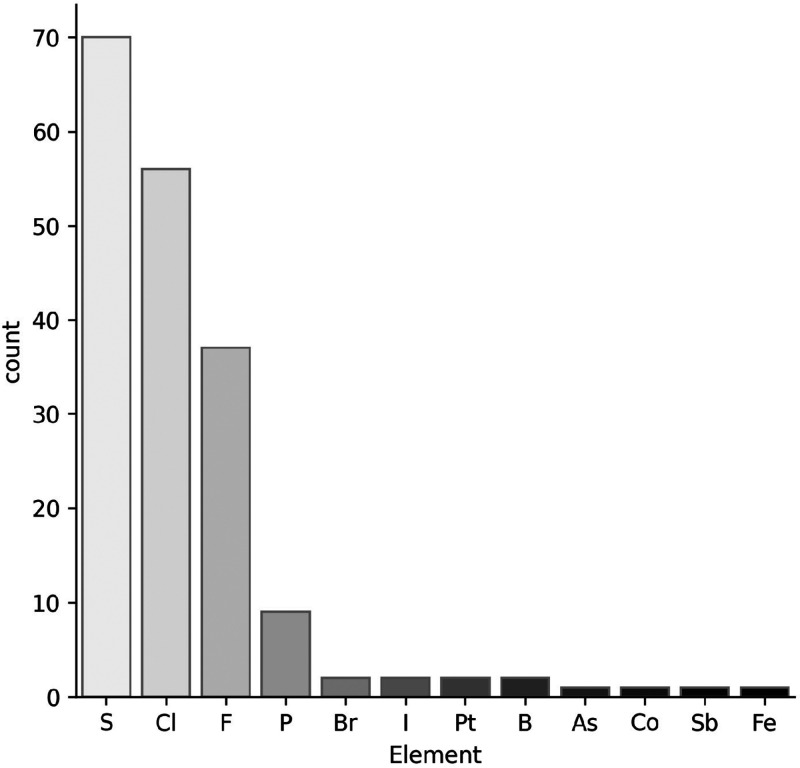
Elements beyond CHON present in EMs.

Taking inspiration from the analysis performed by Smith et al. on a database representing FDA-approved unique small molecules (1086 entries), we analyzed the elements beyond carbon, hydrogen, oxygen, and nitrogen (CHON) present in EMs (Figure 4).^12g^ The most represented element is sulfur as 20% of drugs contain this element, a percentage comparable to the one calculated for FDA-approved unique small molecules (19%). Sulfur is contained almost equally in functional groups (*n* = 40; see below) and in heterocycles (*n* = 33; see below).

While the use of organosulfur compounds dates back ancient times, fluorine makes its entry only in the 1950s, and its frequency in drugs has been gradually rising over the years.^24^ In the same study cited above,^12g^ the impact number for fluorine is reported to be 11%, and the percentage rises to 20% if only drugs approved after 2000 are considered.^12h^ In the EML, the percentage of fluorine is 11%, identical to the one related to the overall FDA-approved drugs.

An opposite trend is observed for chlorine, where the percentage goes from 17% in drugs approved in the 1980s to 10% in drugs approved after 2000.^12g^ Its prevalence for the FDA-approved drugs is 15%, comparable to the EML (16%). The plant kingdom rarely incorporates halogens in its biosynthetic pathways, and this is the reason for which no natural compound displays fluorine, while three EMs contain chlorine, but they originate from sources other than plants (vancomycin, griseofulvin, chloramphenicol).^25^

In the EML, bromine and iodine are each present solely in two drugs. In particular, bromine is found in halothane, the only inhalational anesthetic containing this element, and in bedaquiline, a drug effective in tuberculosis (TB) that was approved in 2012, representing the first new medicine for TB in more than 40 years.^26^ Iodine is present in the antiarrhythmic amiodarone and in the synthetic version of the thyroid hormone levothyroxine.

**Figure 5:**
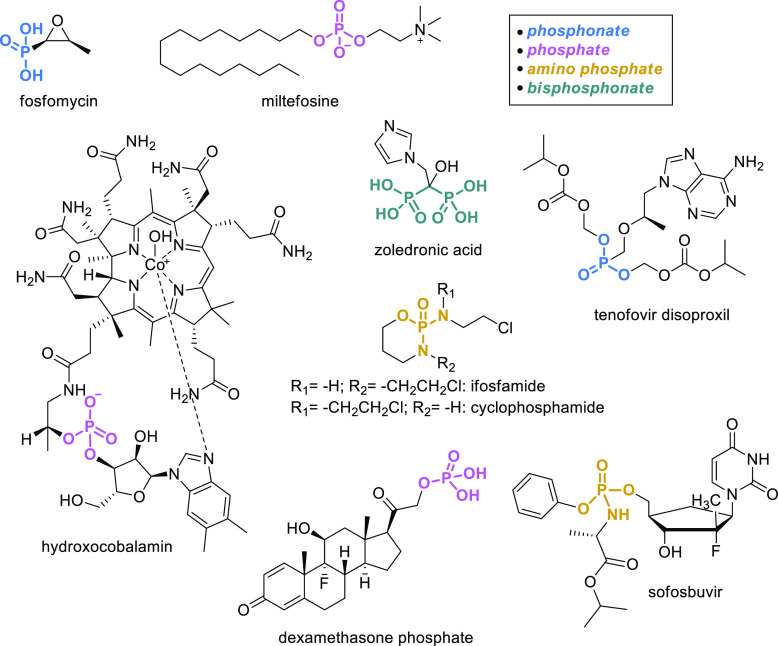
Phosphorus-containing EMs.

Phosphorus is represented in 3% of EM organic drugs (Figure 5): the antibiotic fosfomycin and hydroxocobalamin, the antileishmaniasis miltefosine, and the antiosteoporotic bisphosphonate zoledronic acid. More importantly, phosphorus is often part of prodrug moieties as in the chemotherapeutics ifosfamide and cyclophosphamide, which are both activated to generate the corresponding alkylating nitrogen mustards. Another example is tenofovir disoproxil, a nucleotide analogue reverse-transcriptase inhibitor for the treatment of HIV and HBV. The cleavage of disoproxil releases tenofovir that is phosphorylated to tenofovir diphosphate, the active compound. Similarly, sofosbuvir, an inhibitor of NS5B approved in 2013 for the treatment of HCV, is activated to the corresponding triphosphate by hydrolysis of the carboxylate ester, followed by cleavage of the phosphoramidate and subsequent repeated phosphorylation. Finally, dexamethasone phosphate, in which the phosphate serves to increase water solubility for the oral formulation, is the only example in which the phosphatase-mediated cleavage releases the active moiety that no longer contains phosphorus. Overall, phosphorus is, therefore, present on the list for different purposes and in different chemical forms, as it can be seen in Figure 5.

**Figure 6:**
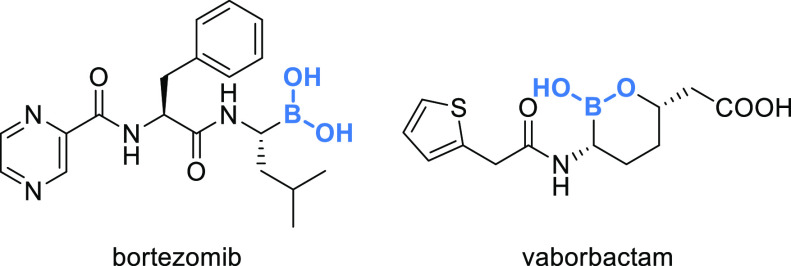
Boron-containing EMs.

Platinum is present in the complexes carboplatin and oxaliplatin, while the boron in the form of boronic acid is a key component in bortezomib, an anticancer drug able to bind the catalytic site of the 26S proteasome and representing the first proteasome inhibitor approved, and in vaborbactam, a nonbeta-lactam beta-lactamase inhibitor (Figure 6). Boron appears to be increasing its presence in drugs, as alongside vaborbactam, approved in 2017 by the FDA; also crisaborole has been recently approved by the FDA, allowing boron to make a definite jump ahead compared with 2014 when the elemental composition of U.S. FDA drugs was first reviewed.^12g^

The remaining elements are arsenic in melarsoprol, antimonium in sodium stibogluconate, cobalt in hydroxocobalamin, and iron in sodium nitroprusside.

##### Which Are the Most Represented Functional Groups and Heterocycles in the EML?

**Figure 7:**
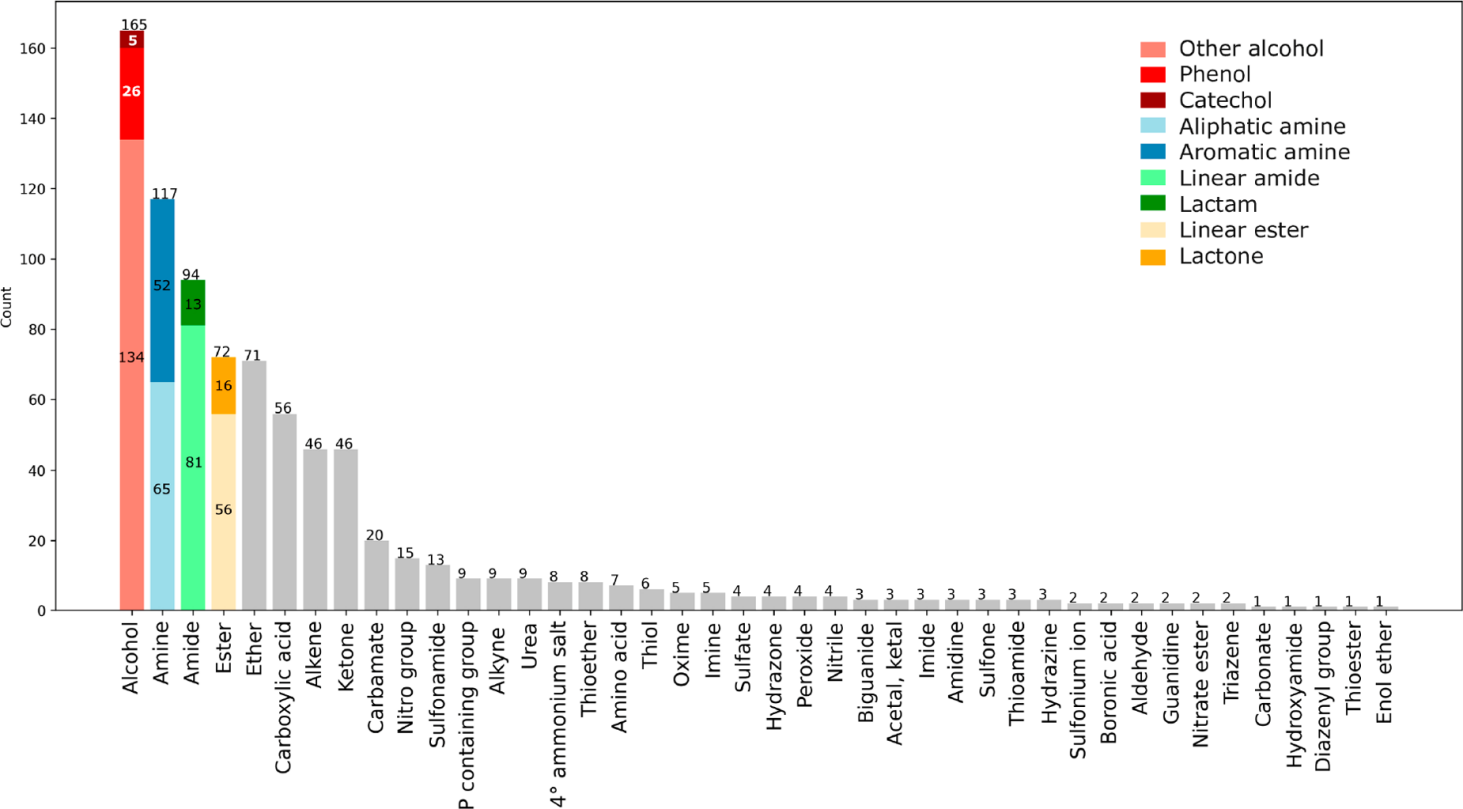
Frequency of selected functional groups found in the EML.

**Figure 8:**
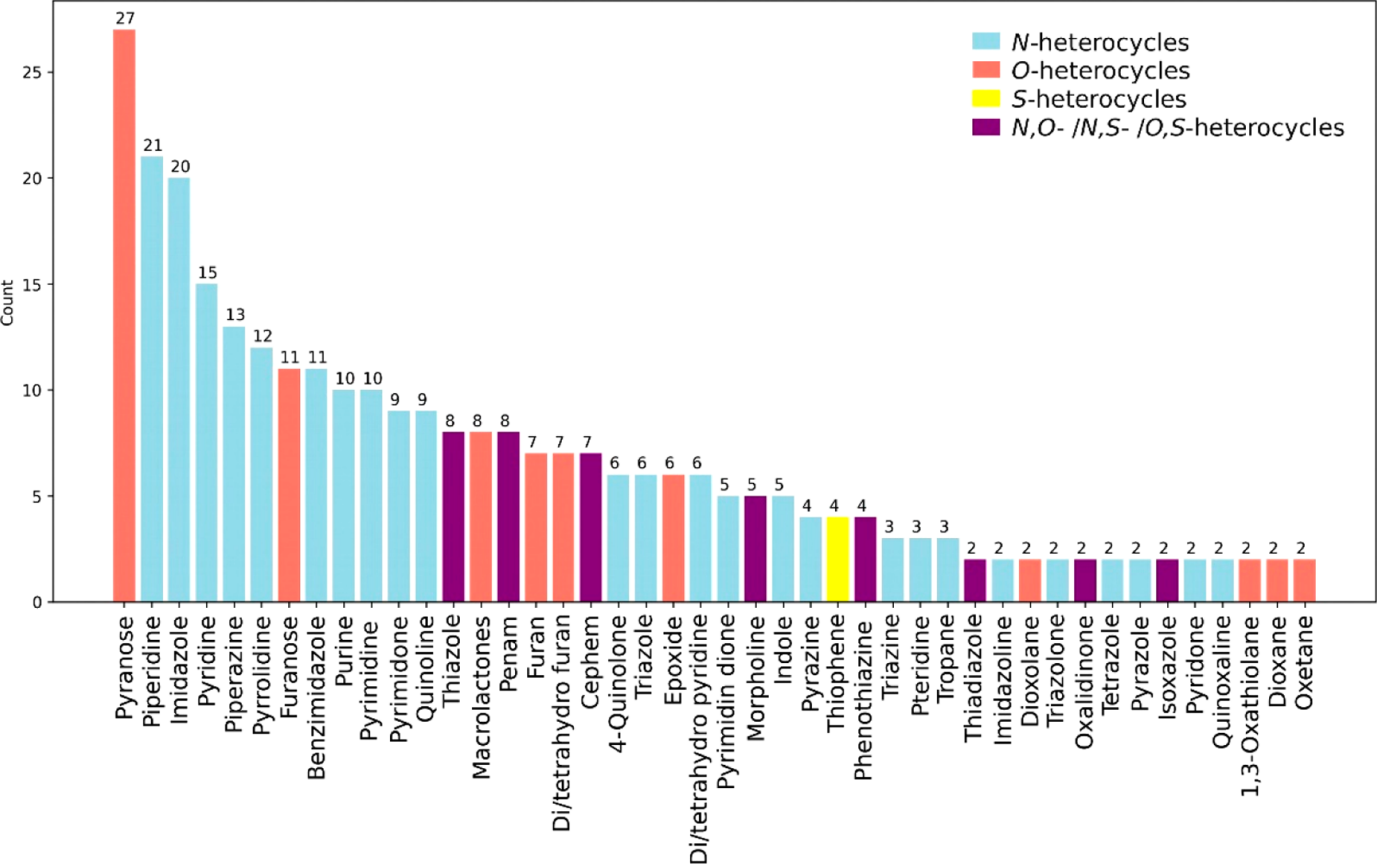
Frequency of heterocycles found in the EML. Heterocycles that are present in more than one EM.^28^

We next analyzed selected functional groups (Figure 7)^27^ or heterocycles (Figure 8) present in EML drugs. For each functional group or heterocycle, we counted the number of EMs containing at least one. We analyzed all the EML drugs by visual inspection, as the use of *in silico* filters proved not to be error-free. Interestingly, in doing this, we found that 15 drugs are macrocycles (with a number of atoms higher than 11 and a maximum of 36 in amphotericin B), 3 drugs contain a medium-size cycle (between 8 and 11 atoms), 25 contain a steroid core, and 26 include sugar moieties.

Alcohol is the most represented functional group (*n* = 165), with almost 50% of EMs displaying at least one hydroxyl moiety. While the catechol substructure is present in only a few drugs (*n* = 5), phenols are more frequent (*n* = 26), and 134 EMs have hydroxyls that cannot be included in the first two categories. Amine is ranked second (*n* = 117), with 65 EMs displaying one aliphatic amine or more and 52 bearing at least one aromatic amine. The third most frequent functional group is the amide (*n* = 94) with 81 EMs that display at least one linear amide and 13 that include at least one lactam.

In fourth place are esters (*n* = 72, either linear *n* = 56 or as a lactone *n* = 16), followed by the ether (*n* = 71). This is quite surprising as esters are usually considered an unconventional functional group, mainly due to their tendency to hydrolyze. Fifteen drugs display more than one ester, with paclitaxel bearing the maximum number of this group (*n* = 4). Excluding the 19 drugs that are present with an INNM (signifying that the ester is rapidly cleaved to release the active moiety), the remaining 50 drugs were analyzed to identify which of them are active as such and which are prodrugs that need to be metabolized to give active moieties. We found that only 7 are prodrugs (e.g., simvastatin, oseltamivir, enalapril, latanoprost, misoprostol, valganciclovir, sofosbuvir), while the remaining 43 esters are an intrinsic feature of the active principle. In this respect, it is interesting to note that the structure of these esters is heterogeneous, spanning from soft drugs in which the cleavage of the nonhindered linear ester guarantees a short activity (e.g., suxamethonium, atracurium) to more hindered esters where the functional group is resistant to hydrolysis (alfa-substituted, erythromycin; alfa-unsaturated–natamycin; endocyclic etoposide; bound to cycles, permethrin). As expected, since the ester moiety is rarely inserted on purpose, only 6% of de novo drugs contain this functional group, which is instead more abundant in the natural (22%) and nature-inspired (33%) EMs.

The most represented *O*-heterocycles in the EML are pyranose (*n* = 27), furanose (*n* = 11), and macrolactone (*n* = 8), a frequency also shared by FDA-approved drugs^12c^ (salmon bars in Figure 8). The fourth position is taken by furan (*n* = 7) and its hydro forms (*n* = 7), structures that, percentage-wise, are less represented in the FDA-approved drugs, followed by epoxide that occurs in 6 EMs.

Regarding *N*-heterocycles, the most represented substructures are piperidine (*n* = 21), imidazole (*n* = 20), pyridine (*n* = 15), piperazine (*n* = 13), and pyrrolidine (*n* = 12), a situation that is reflected in FDA-approved drugs,^12f^ except for imidazole, that is not as abundant in the latter (light blue bars in Figure 8).

##### PAINS

PAINS (pan-assay interference compounds) is a concept introduced in 2010 by J. B. Baell and G. A. Holloway that coined this acronym to indicate those classes of compounds defined by a common substructural motif that is responsible for an increased chance of any member emerging as a hit in any given assay.^29^ Despite not being a black-and-white issue, PAINS can be recognized by electronic filters that help the medicinal chemist in the identification of those compounds that have a high possibility of giving anomalous screening results. PAINS do not include known toxicophorics or aggregate-forming molecules but refer to compounds that interfere with the target or with the assay setup and methodology.^30^

**Figure 9:**
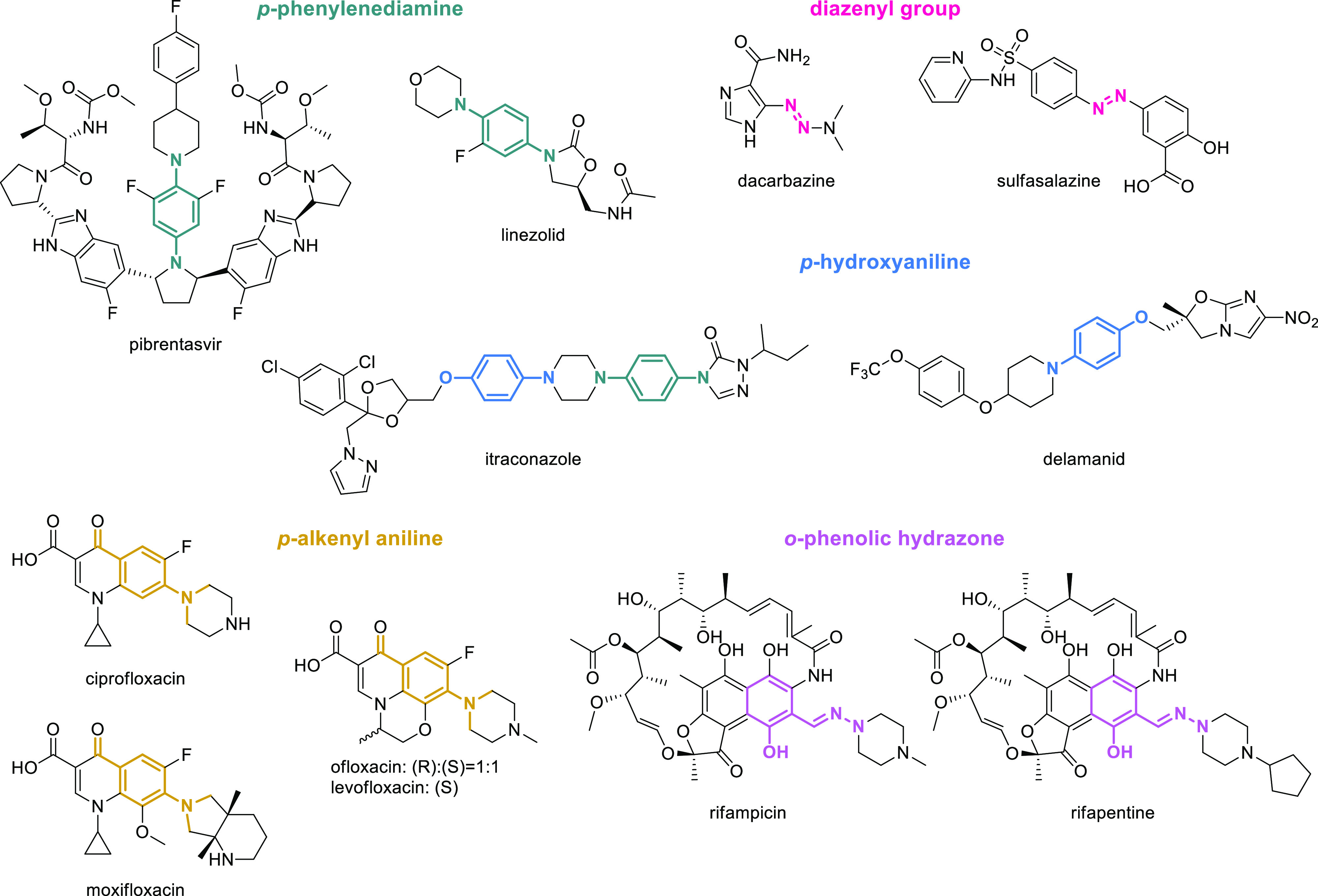
Structures of EMs displaying PAINS.

The original suggested PAINS substructures were numerous (Tables S6, S7, and S9 in the Supporting Information from the work of Baell and Holloway).^29^ As an academic exercise, we evaluated how many of these were found in EMs by using the FILTER software from OpenEye^31^ and found that only 12 drugs contained PAINS cores (Figure 9). In 2018, J. B. Baell and J. W. M. Nissink published a second optimized set of the 13 most highly populated and generally recognized PAINS substructures.^30^ All 12 drugs have PAINS chemotypes that belong to this shorter list, strengthening the notion that this set may be sufficient for PAINS recognition. The percentage of PAINS-containing drugs in the EML is 3%, slightly lower compared with the one reported for FDA-approved drugs (5%).^30^ It should be noticed that the conversion of the original filters in SMARTS may not lead to entirely accurate calls, as is the case of ofloxacin and its analogues.

##### Structural Alerts

Structural alerts (also known as toxicophorics) are functional groups or substructures that are associated with idiosyncratic adverse drug reactions (IADRs) that usually affect liver, skin, and/or blood.^32^ The toxicity is unrelated to the pharmacological action of the drug but is usually related to its metabolism into electrophilic reactive metabolites that covalently modify host proteins.^33^ Starting from the seminal list reported by Nelson, the number of structural alerts has been implemented over the years, reaching hundreds of toxicophores, with the aim of minimizing the attrition related to the toxicity of drug candidates.^33^ In a retrospective analysis, structural alerts were found in 55 out of 68 drugs that were either withdrawn due to IADRs or had black box warnings (BBW), suggesting that these alerts may be prognostic.^34^ On the other hand, it must be said that numerous drugs that contain structural alerts are devoid of IADRs, suggesting a need for a cautious evaluation of this aspect in discarding potential drug candidates.

**Figure 10:**
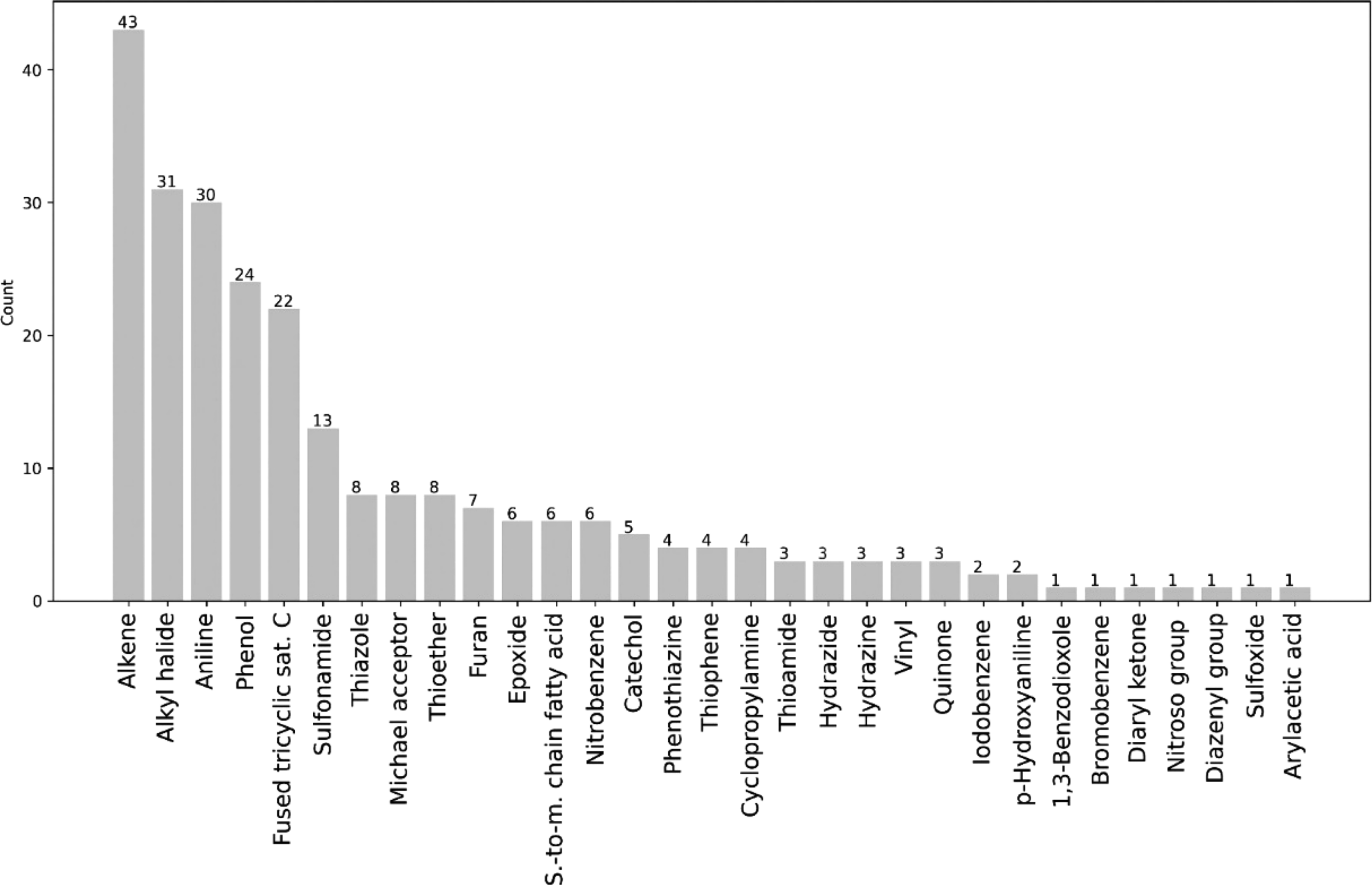
Represented toxicophorics in the EML. Short-to-medium (s-to-m) chain fatty acids are carboxylic acids bound to a carbon chain with more than 3 atoms.

Instead of considering the hundreds of structures described in the literature as toxicophores, we have opted to refer to two focused lists that contain the most significant alerts and that partially overlap. The first one includes the structural alerts found in drugs withdrawn due to IADRs,^34^ while the second set of alerts was built by retrieving hepatotoxic small molecules from LiverTox, a U.S. NIH online resource for information on human liver injuries induced by drugs.^35^ These two lists were further implemented with other well-known toxicophorics (e.g., Michael acceptor, epoxide, vinyl, quinone). We then analyzed by visual inspection our database of EMs, and Figure 10 lists all toxicophorics found.

Some toxicophorics are rare or absent^36^ in the EMs, while others are recurring (e.g., alkene, alkyl halide, aniline, phenol). The presence of some structural alerts in a high percentage of EMs in part questions the need to refine the toxicophoric list, also putting the single moieties in context. For example, at face value, alkenes are present in 12% of EMs. Yet, the toxic potential of this functional group is significantly higher in lipophilic drugs (that are more prone to CYP-mediated oxidation to epoxides) compared to hydrophilic compounds (that are less prone to oxidative metabolism).

**Figure 11:**
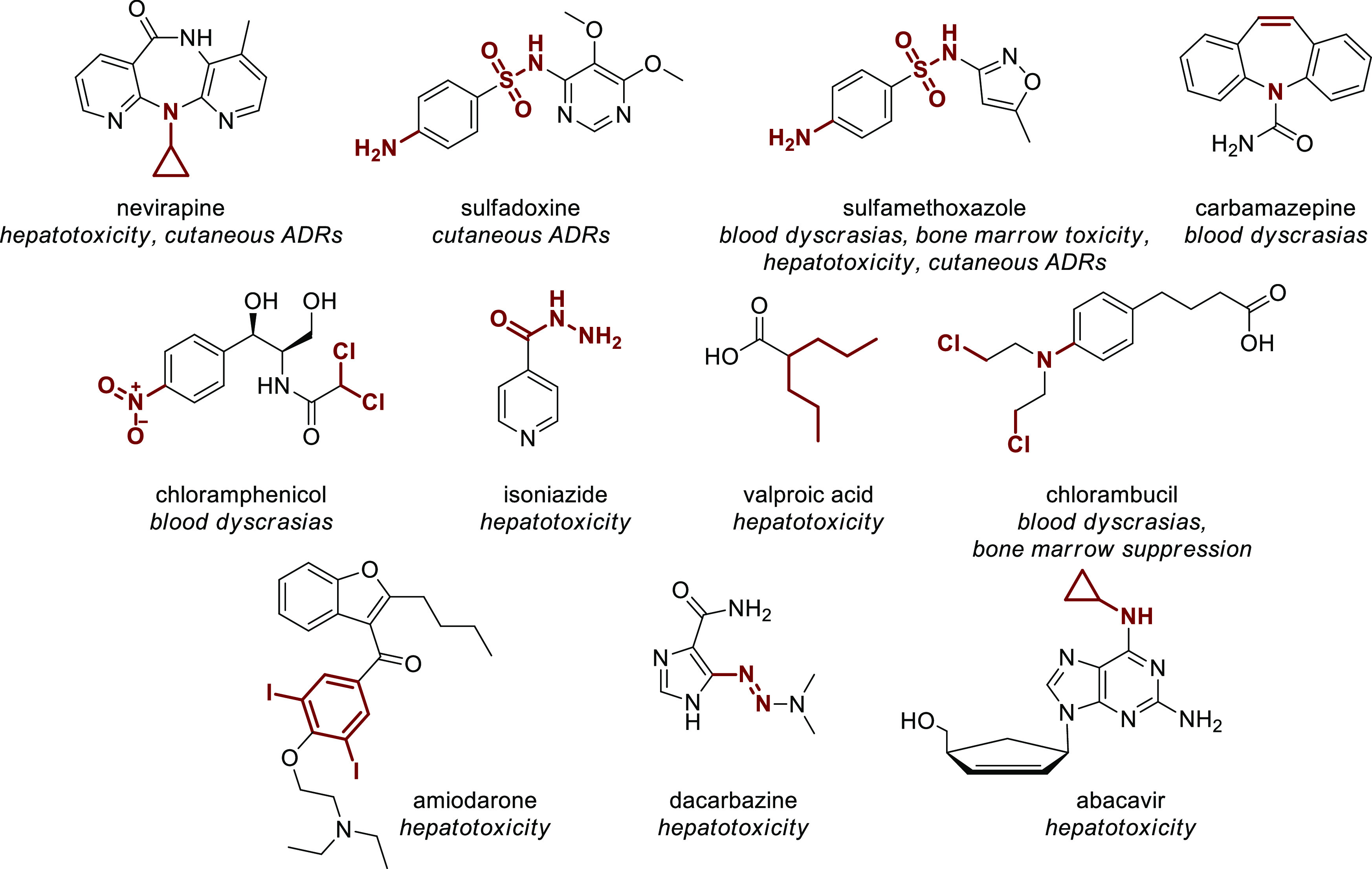
EMs containing a structural alert associated with a BBW due to IADRs. Structural alerts are highlighted in red.

To the best of our knowledge, in the EML, there are at least 16 drugs associated with a BBW due to IADRs, and 11 out of 16 contain one or more structural alerts (Figure 11). Among the two most represented structural alerts on the EML, alkenes are not overrepresented in the BBW drugs (9% compared with 13% on the EML), while alkyl halides might be (18% compared with 9%). Highly overrepresented toxicoforic structures in BBW drugs are aniline (3 out of 30 on the list), sulfonamide (2 out of 13), and cyclopropylamine (2 out of 4), the latter associated in both cases to hepatotoxicity, pointing to these substructures as a real concern in drug research and development.

#### The Importance of Chirality in the EML

Since 1984, when the theory of E. J. Ariëns was first enunciated,^37^ the chirality of drugs has begun emerging as an important aspect in early-stage development. To investigate this aspect, we evaluated the chiral nature of EMs according to the intrinsic nature of the molecules (de novo vs nature-inspired vs natural).

Thirty-seven percent of all the organic EMs is represented by achiral compounds, and not surprisingly since it is known that nature is chiral, 67% of them belong to the de novo subcategory (*n* = 84), only 10% to the nature (*n* = 13), and the remaining 23% to the nature-inspired (*n* = 29). Among the remaining chiral products (*n* = 219, 63%), 169 compounds bear more than one stereocenter, and the majority are nature-inspired (*n* = 88) or natural (*n* = 50) drugs, while de novo compounds that display more than one stereocenters are less (*n* = 31) (Table 2).

**Table 2:** Chirality Profile of EMs

	all organic EMs (*n* = 345)			
	all, *n* (%)	de novo, *n* (*n* = 143)	nature-inspired, *n* (*n* = 132)	natural, *n* (*n* = 70)
achiral compounds	126 (37^a^)	84	29	13
chiral compounds	219 (63^a^)	59	103	57
1 stereocenter	50 (23^b^)	28	15	7
>1 stereocenter	169 (77^b^)	31	88	50
mixture of stereoisomers	18 (8^b^)	11	5	2
single stereoisomer	201 (92^b^)	48	98	55

a % of compounds on a total of 345 active principles included in the EML.

b % of compounds on a total of 219 chiral compounds.

**Figure 12:**
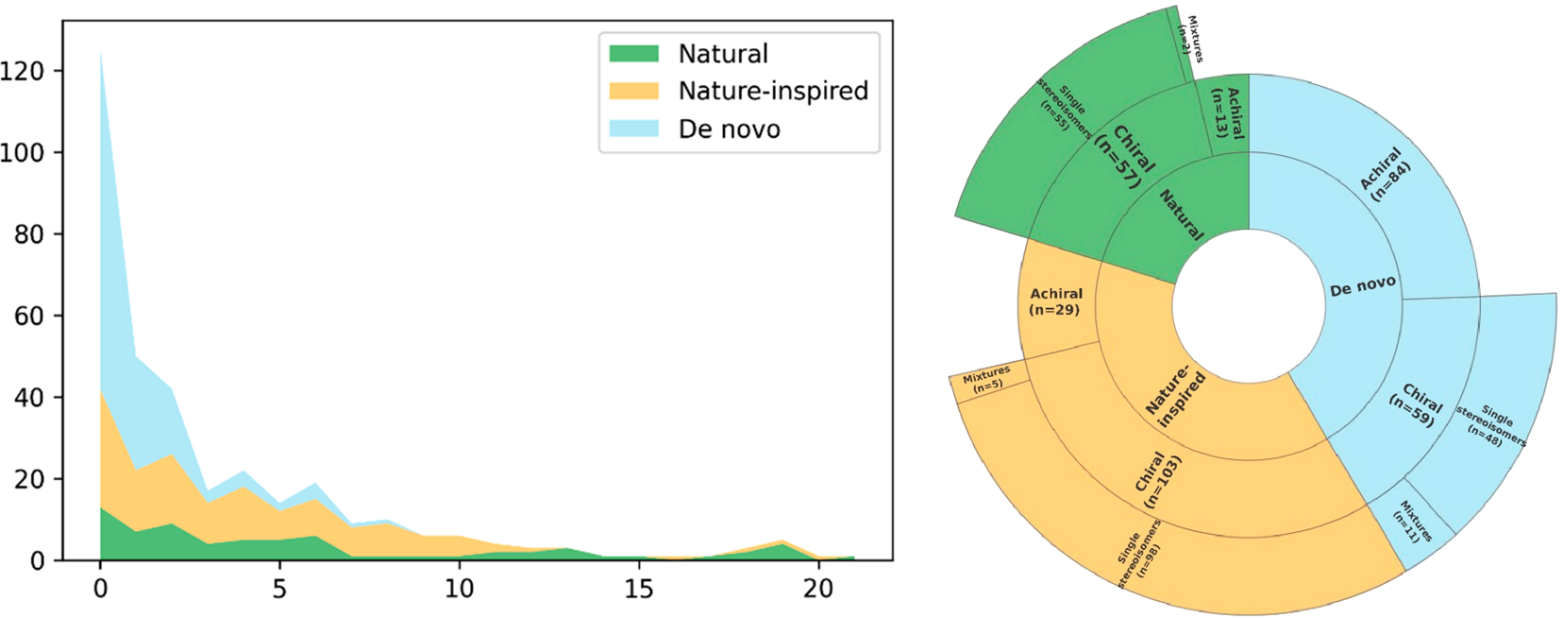
Distribution of chiral centers in de novo, nature-inspired, and natural drugs. A more detailed version of Figure 12 is present in the Supporting Information.

**Figure 13:**
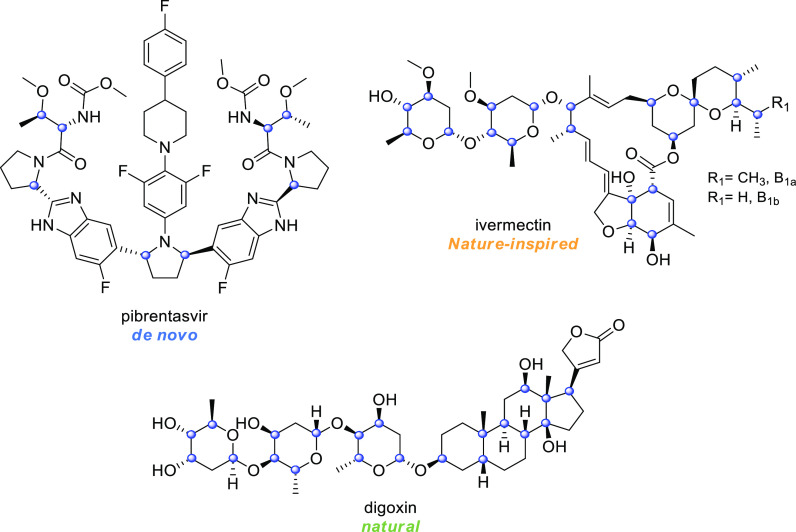
Structures of the de novo, nature-inspired, and natural drugs that display the highest amount of stereocenters. Stereocenters are highlighted with a blue circle.

The distribution of the chiral centers among the three subcategories reveals that the majority of de novo drugs display one to six chiral centers (Figure 12), with only one compound bearing 8 stereocenters (that is pibrentasvir, Figure 13). On the contrary, the distribution of chiral centers in the natural and nature-inspired categories is similar, with 24 drugs bearing more than ten chiral centers (Figure 12). The drug that contains the highest number of stereocenters is digoxin with 21 chiral centers, followed by ivermectin that displays 20 stereocenters (Figure 13).

Only 8% (*n* = 18) of the chiral EMs is licensed and sold as a mixture of stereoisomers (that we referred to as racemates), while the remaining 201 drugs are approved as single stereoisomers.

Racemates have gone out of fashion in the 1990s,^38^ when the FDA published guidelines for the development of chiral drugs. We, therefore, scrutinized the year of approval by the FDA and found that 6 out of 18 racemates were approved after the guidelines were issued. Yet, when the search was extended worldwide, we found that, out of the 18 racemates, 17 were indeed approved before 1992, suggesting that the guidelines had a deep impact on drug development. The only exception is represented by thalidomide that was approved in 1998 with the novel therapeutic indication for multiple myeloma and, since the drug undergoes a well-known *in vivo* racemization, it cannot be developed as a single enantiomer.

It should be considered that a number of compounds have undergone a chiral switching strategy in the previous decades,^39^ which were originally developed as racemates but, after the approval of the racemic ancestor, the single preferred stereoisomer, named eutomer, was developed as a new molecular entity. In the case of the EML, it is possible that the eutomer derived from chiral switching is included in the square boxes that we have not considered in our analyses. For example, the best-known case of chiral switching is represented by the eutomer esomeprazole, but only its racemic ancestor omeprazole is listed in the EML, while esomeprazole is presumably included in the square box. Yet, this most likely means that the EML Expert Panel did not consider chiral switches of a significant clinical added benefit, in a dissimilar fashion compared with the market recognition that some of these molecules received in the 1990s (esomeprazole, escitalopram). Interestingly, the list contains both the racemate ofloxacin and the eutomer levofloxacin, which is the only EM resulting from chiral switching.

#### Assessment of Absorption for Oral Drugs

Different authors have set rules to increase the likelihood of high oral absorption and to reduce failure due to poor pharmacokinetics (PKs). Among all the rules reported in the past, Lipinski’s rule,^40^ that dates back to 1997, still represents the best-known rule of thumb.

While in 1991 the attrition due to poor availability and PK was responsible for 39% of failures in clinical studies, by 2000, the percentage was reduced to 8%, with a concomitant increase of attrition due to toxicity (19%).^41^

**Figure 14:**
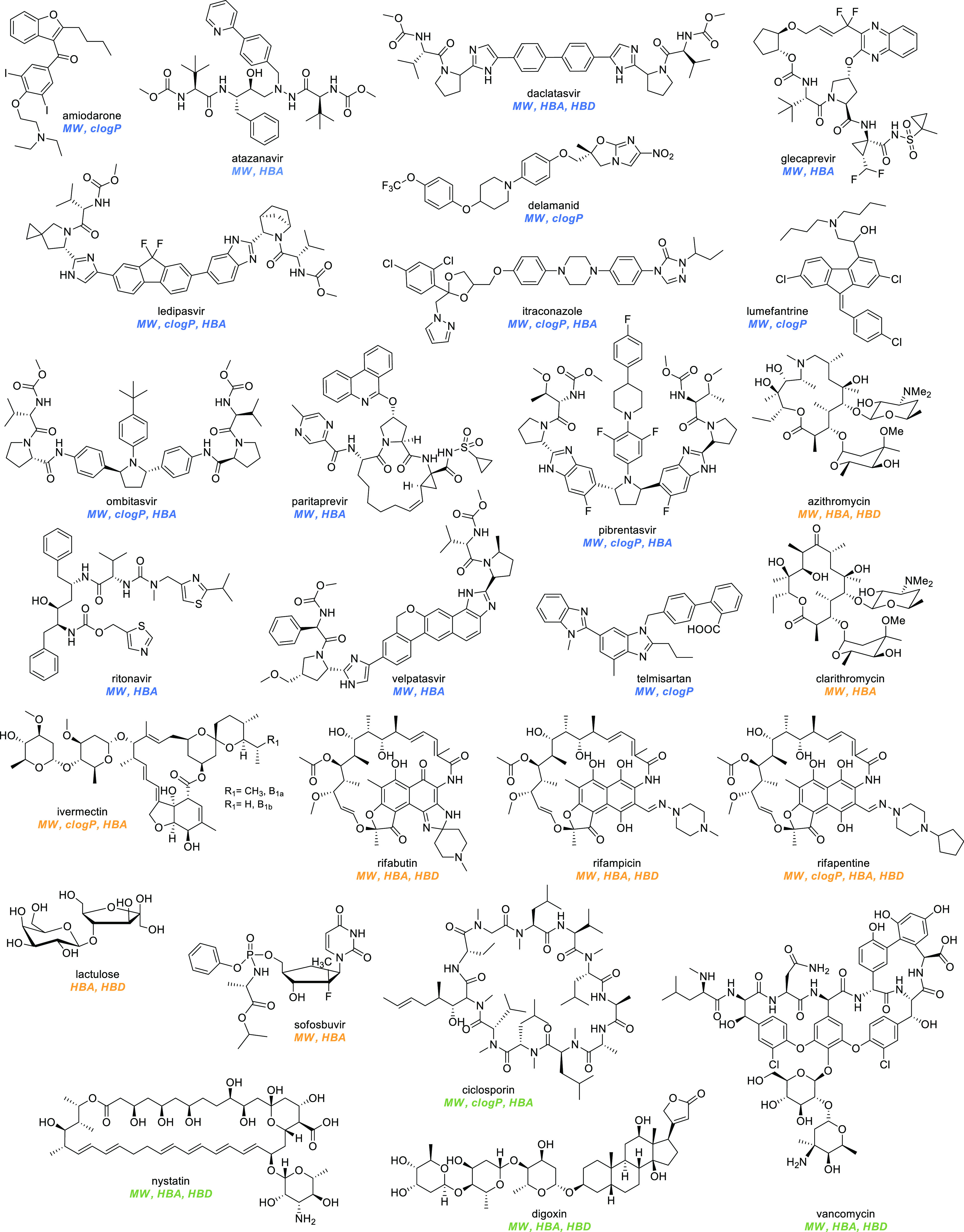
Structures of oral EMs that violate two or more of Lipinski’s parameters. Abbreviations: clog*P*, calculated log*P*; HBA, hydrogen bond acceptor; HBD, hydrogen bond donor; MW, molecular weight; clog*P* were extracted using DrugBank^21^ or ChemSpider^42^ databases.

We have analyzed the properties of oral EMs (Table 3) and found that the majority of compounds obeys to the Lipinski’s rule of five. The percentage of EMs that violate two or more parameters is 14% (*n* = 31), compared to the 6% of all FDA-approved drugs.^43^ The violations in order of occurrence are molecular weight (MW) > clog*P* = hydrogen bond acceptors (HBAs) > hydrogen bond donors (HBDs). While we were expecting to find most of the compounds that fall outside the Lipinski’s rule in the natural category, no significant differences can be found among the three subsets of molecules (DN 13%; NI 16%; N 14%). It should be noticed that among nature-inspired drugs, 5 compounds are prodrugs that are metabolized to an active species that obeys Lipinski’s rule (e.g., chloramphenicol palmitate, clindamycin palmitate, retinol palmitate, tenofovir disoproxil, dabigatran etexilate) and for this reason are not displayed in Figure 14, in which the remaining 26 molecules are illustrated. Moreover, among the de novo drugs, there is a populated cluster of antiviral compounds (9 out of 14) that account for most of the violations (e.g., anti-HIV, atazanavir; anti-HCV, daclatasvir, ledipasvir that are believed to take advantage of uptake carriers).^44^ Interestingly, 10 of the 31 drugs that have at least two violations have been developed in the last two decades, suggesting that drug development is moving away from a strict risk assessment of oral absorption.

**Table 3:** Properties of Oral EMs^a^

	oral organic EMs (*n* = 217)			
	all, *n* (*n* = 217)	de novo, *n* (*n* = 108)	nature-inspired, *n* (*n* = 80)	natural, *n* (*n* = 29)
MW ≤ 500 Da	179	87	69	24
clog*P* ≤ 5	189	95	70	24
HBAs ≤ 10	188	96	68	24
HBDs ≤ 5	208	107	75	26
0 or 1 violations	186	94	67	25
2 or more violations	31	14	13	4

a Abbreviations: clog*P*, calculated log*P*; HBA, hydrogen bond acceptor; HBD, hydrogen bond donor; MW, molecular weight; clog*P* were extracted using DrugBank^21^ or ChemSpider^42^ databases.

#### Topical Cutaneous Drugs

In the EML, 3 compounds display a transdermal route of administration, while 17 are administered through a cutaneous route. Among the latter, 4 of them are antiseptics or disinfectants (chlorhexidine, chloroxylenol, ethanol, glutaral), for which no absorption is needed, and therefore they have not been considered for this analysis. Four cutaneous EMs out of the remaining 13 are listed as salts (e.g., lidocaine chloride, miconazole nitrate, mupirocin calcium, terbinafine chloride). Paracellular transport is the most important pathway exploited by therapeutic agents to penetrate the skin, and the main chemical feature that may predict absorption is a low MW (<500 Da).^45^ None of the 13 EMs falls outside the rule of 500 Da. It is interesting to note that, if a transdermal absorption is required, the molecular weight criterium becomes even stricter and lowers to 350 Da, and indeed, if we consider the transdermal EMs (scopolamine, fentanyl, nicotine), this condition is fulfilled.

High lipophilicity (1 > log*P* < 4)^46^ and low melting point (<200 °C)^47^ are also said to determine the extent of absorption through the skin. Three of the 13 cutaneous drugs display a negative log*P* value (e.g., acyclovir, fluorouracil, urea) and 3 of them a log*P* above 5 (e.g., miconazole, permethrin, terbinafine), while only 7 drugs (54%) fall in the log*P* range 1–4. Furthermore, only 8 compounds (62%) out of 13 exhibit a melting point below 200 °C, questioning the absolute validity of these requirements (for the full set of properties of cutaneous drugs, see Supporting Information). It should be nonetheless acknowledged that those compounds that fall outside this condition, especially when very polar, might use the skin appendage pathway, a type of transport that relies on hair follicles and sweat ducts.^48^

#### Injectable Drugs

Injectable drugs account for 42% of all the organic products (*n* = 145) in the EML. Eighty-eight of them are included exclusively as injectables in the EML, while the remaining 57 are also included as oral forms. It is possible, though, that some of the pure injectable drugs might be marketed in further formulations outside the EML.

Fifty-three percent of the natural compounds are injectables, compared with 48% of the nature-inspired and only 30% of the de novo drugs. This distribution might be explained, considering that many de novo drugs are rationally designed to assess their likeness to be orally absorbed prior to synthesis. Natural products that are solely listed as injectable (*n* = 28) show MW on average higher (50% over 500 Da) compared with nature-inspired (32%) and de novo (16%) injectable drugs.

Solubility is far more important in the case of injective administration, and an obvious strategy to improve this property is salt formation. Not surprisingly, the percentage of all the injectable drugs (*n* = 145) administered in the salified form (*n* = 66, 46%) is higher compared with oral salified drugs (*n* = 63, 29%). Besides salt formation, prodrug design might also help in increasing solubility, and indeed, 11 injectable drugs are prodrugs listed with an INNM (e.g., chloramphenicol sodium succinate, dexamethasone sodium phosphate). On the other hand, substructures such as decanoate, palmitate, or enanthate are usually inserted to provide prodrugs with prolonged release (e.g., fluphenazine decanoate, testosterone enanthate).

#### How Many Organic EMs Cross the Blood–Brain Barrier? The BBB Score

The blood–brain barrier (BBB) is a dynamic structure that acts as a physical and selective barrier aimed at finely regulating the transport of molecules from the bloodstream to the central nervous system (CNS) and vice versa.^49^ This formidable “gatekeeper” impedes the entrance in the brain of around 98% of systemically administered molecular entities and of nearly all biological drugs.^50^ Hence, on the one hand, BBB represents a stumbling block for drugs specifically designed for the treatment of neurological conditions, while, on the other, it limits the risk of undesired neurological toxicities for non-CNS drugs.

Following Lipinski’s rule, a set of guidelines to predict BBB permeability have been proposed that take into account physicochemical properties such as MW, hydrogen bonds, and polar surface area (PSA).^51^ Subsequently, many efforts have been focused in the development of algorithms able to accurately measure and predict molecule penetration across the BBB^52^ Among these, the BBB score, designed by Gupta and Colleagues in 2019, stands out as the one with the highest sensibility and sensitivity in determining whether a drug can be potentially delivered to the CNS.^52g^ Briefly, the BBB score takes into account (i) the number of aromatic rings (AR); (ii) the number of heavy atoms (HA); (iii) MWHBN, composed of the number of HBD atoms, the number of HBA atoms and the MW; (iv) the topological PSA; (v) the p*K*_a_ at physiological pH. The BBB score ranges from 0 to 6, and drugs attaining ≥4 points are identified as highly likely to reach the CNS.

The five physicochemical descriptors included in the BBB score were estimated for each organic EM according to the model proposed by Gupta and colleagues.^52g^ In the case of INNM drugs indicating prodrugs, the BBB score was calculated on the active moiety (diloxanide furoate, fluphenazine decanoate, and enanthate). Briefly, AR and HA were calculated using ChemAxon,^53^ while MW, HBA, HBD, and topological PSA were assessed through OpenEye.^31^ The MWHBN descriptor (HBD+HBA)/√MW) was calculated as previously described.^52g^

**Figure 15:**
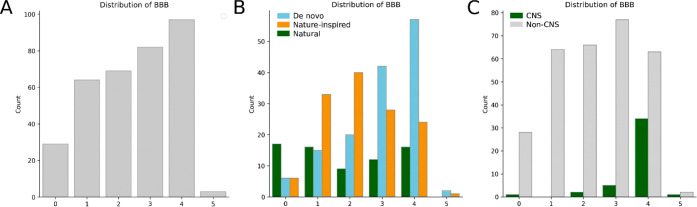
(A) BBB scores in organic EMs, (B) stratified for their origin, and (C) CNS vs non-CNS drugs.

The distribution of BBB scores in our set of organic EMs is illustrated in Figure 15A. The majority of EMs (*n* = 244, 71%) fall in the score range of 0–3.99 (class 0–3), thus showing a low potential of crossing the BBB. The remaining drugs (29%) have a BBB score ≥4 and, hence, are plausibly able to reach the CNS. Among the latter, the vast majority of molecules are de novo drugs (*n* = 59, 59%), followed by those classified as nature-inspired (*n* = 25, 25%) and natural (*n* = 16, 16%, Figure 15B). Conversely, nature-inspired and natural drugs belong mainly to the set of molecules with a BBB score <4. Natural EMs are the most present drugs in the lowest BBB score class (score range: 0.00–0.99; *n* = 17, 59%). Lastly, as expected, CNS drugs, that is, EMs with an ATC first-level code N, have a significantly higher possibility of having a BBB score above 4 (Figure 15C). Nevertheless, 8 CNS drugs (18%; levodopa, carbidopa, isoflurane, halothane, lamotrigine, ethosuximide, valproic acid, phenobarbital) have a BBB score below 4. Obviously, levodopa enters the CNS through a carrier for amino acids, and carbidopa does not cross the BBB, thereby explaining their presence in the low category.^54^ Similarly, valproic acid is taken up into the brain via a transport system for fatty acids.^55^ Nonetheless, the presence of other drugs, including isoflurane and halothane, is unexplained, further consolidating the reported ability of these general aesthetics in altering the BBB permeability to diffusional processes.^56^

Last, it is well-known that CNS drugs tend to contain at least one basic functionality, as positive charges favorably interact with the negatively charged head groups of phospholipids at the BBB, assisting BBB influx. As expected, 57% of the CNS drugs are basic, 25% are neutral (including one amphotericdrug, that is levodopa), and only 18% are acids. Eighty-four percent of all of the basic CNS drugs are represented by amines, and this recurrence is ascribable to the fact that out of 21 amine-displaying EMs, 14 target GPCRs that are known to have a preference for amine groups.^57^ Among amines, tertiary amines are the most represented (90%), and secondary amines are less abundant (10%), while no primary amine is present. Different factors play a role in determining this scenario, and desolvation penalty, which is far less significant for tertiary amines, is one of them.

## Final Remarks and Conclusions

The EML is an essential tool across all nations to choose the medicines that give the greatest magnitude of benefit to global health. Nowadays, it is intended to be an aid to low- to medium-resource countries as well as resourceful countries.

The present review was aimed at understanding the medicinal chemistry of these drugs through a number of systematic analyses. A limit of the analysis is the fact that, when a drug is listed with a square box, other drugs with similar efficacy are, by definition, excluded from the analyses. Unfortunately, the full list of drugs included in the square box is not publicly available and, therefore, cannot be analyzed. It should be noticed that there are a number of different reasons for listing one compound over another, thereby creating an uncontrollable bias in the analyses.

The present review did not focus specifically on the differences in medicinal chemistry between this data set and others, for example, the FDA-approved drugs, which is often the object of similar analyses. While this would have been interesting, it must be acknowledged that this data set is smaller, selected, and differs significantly in the ATC code composition and year of approval, thereby making a meaningful comparison impossible. The presence of the square box also makes the two data sets not comparable.

Notwithstanding these limitations, the analyses presented here show something that was most likely obvious. There are no rules regarding drugs that cannot be broken, and the rules only serve to increase chances that a particular molecule may become a drug: there are essential medicines that have 21 stereocenters and still are at the fore-front of heart failure treatment, that are administered *per os* but fail 4 Lipinski’s requirements out of 4, that include multiple structural alerts but are reasonably safe, and that are predicted not to cross the BBB but are among the most widely used general anesthetics. These are not only drugs that have made it to the market but are drugs that make a difference to global health.

One of the objectives of the present review is to disseminate the presence of the EML, which may also be used for teaching and educational purposes. In a pharmaceutical world of ever-increasing complexity in which new drugs arrive on the market every week, the EML may be a good starting point to determine what is essential to teach and to draw examples.

More importantly, this review is intended to remind those involved in pharmaceutical R&D that not all drugs are equal, and such a concept should be kept in mind also in research, while, at times, we feel that our greatest dream is to bring a drug on the market, a bigger dream may be to develop a drug that will make it on the Essential Medicines List.

## Supporting Information Available

The DT network of all approved drugs and of EMs; distribution of chiral centers in the EMs; properties of cutaneous EMs; data set of EMs (PDF)

## Abbreviations Used

ADR: adverse drug reaction
AR: aromatic ring
ATC: Anatomical Therapeutic Chemical Classification System
BBB: blood–brain barrier
BBW: black box warning
Bcl-2: B-cell lymphoma 2
CHON: carbon, hydrogen, oxygen, and nitrogen
clog*P*: calculated log*P*
CML: chronic myeloid leukemia
CNS: central nervous system
DN: de novo
DT: drug-target
EM: essential medicine
EML: Essential Medicines List
EMA: European Medicines Agency
FDA: Food and Drug Administration
GABA: γ-aminobutyric acid
HA: heavy atom
HBA: hydrogen bond acceptor
HBD: hydrogen bond donor
HBV: hepatitis B virus
HCV: hepatitis C virus
HIV: human immunodeficiency virus
IADR: idiosyncratic adverse drug reaction
INN: international nonproprietary name
INNM: international nonproprietary name modified
MW: molecular weight
N: natural
NI: nature-inspired
NIH: National Institutes of Health
NS5B: nonstructural protein 5B
PAINS: pan-assay interference compounds
PK: pharmacokinetic
PSA: polar surface area
s-to-m: short-to-medium
TB: tuberculosis
U.S.: United States
WHO: World Health Organization
