# Supplementary material for: Essential Medicinal Chemistry of Essential Medicines
Source: J Med Chem. 2020 Apr 30;63(18):10170–87. doi: 10.1021/acs.jmedchem.0c00415 (PMC8007110; doi:10.1021/acs.jmedchem.0c00415)
Supplement: Supplementary file 1 — jm0c00415_si_001.pdf [file jm0c00415_si_001.pdf]

Supporting Information

The Essential Medicinal Chemistry of Essential Medicines

Marta Serafini,‡ Sarah Cargnin,‡ Alberto Massarotti, Tracey Pirali*, and Armando A. Genazzani

Department of Pharmaceutical Sciences, Università del Piemonte Orientale, Largo Donegani 2, 28100 Novara, Italy

Table of contents

p. S2 The drug–target (DT) network of all approved drugs.

p. S4 The drug–target (DT) network of EMs.

p. S6 Distribution of chiral centers in the EMs.

p. S7 Properties of cutaneous EMs.

p. S8 Classification of EMs.

p. S26 References.

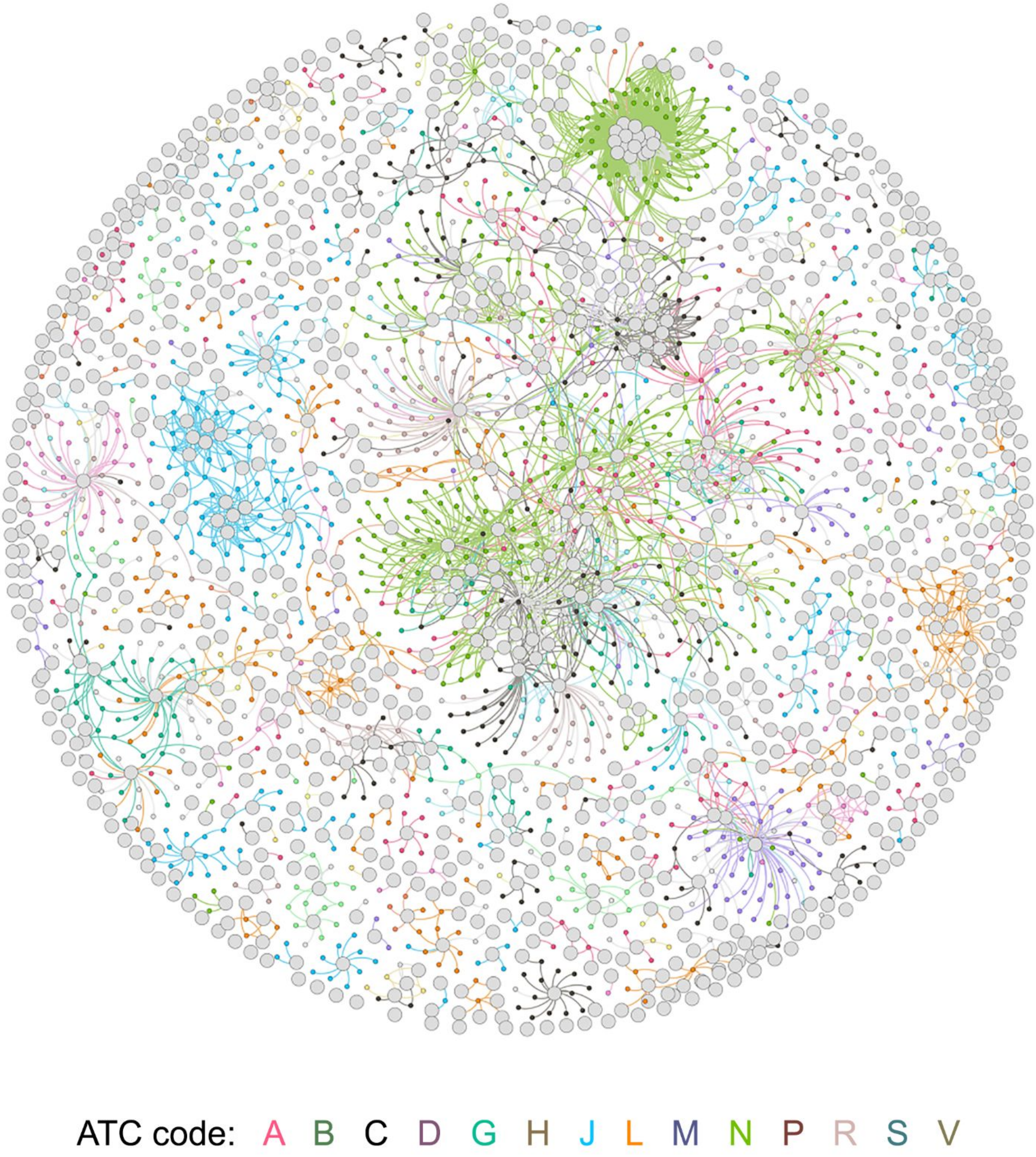
ATC code: A B C D G H J L M N P R S V

Figure S1. The drug–target (DT) network of all approved drugs. The DT network is generated by using the known associations between drugs and targets extracted from the DrugBank database.[1] As of March 2, 2020, DrugBank (version 5.1.5, released 2020-01-03) includes 2635 approved small molecule drugs and 1367 approved biologics. Additionally, 1148 non-redundant proteins (*i.e.* drug target/enzyme/transporter/carrier) sequences are linked to these drug entries. Small and big circles correspond to drugs and target proteins, respectively. A link is placed between a drug node and a target node if the protein is a known target of that drug. Drug nodes and connecting links are colored according to the ATC code of the drug.

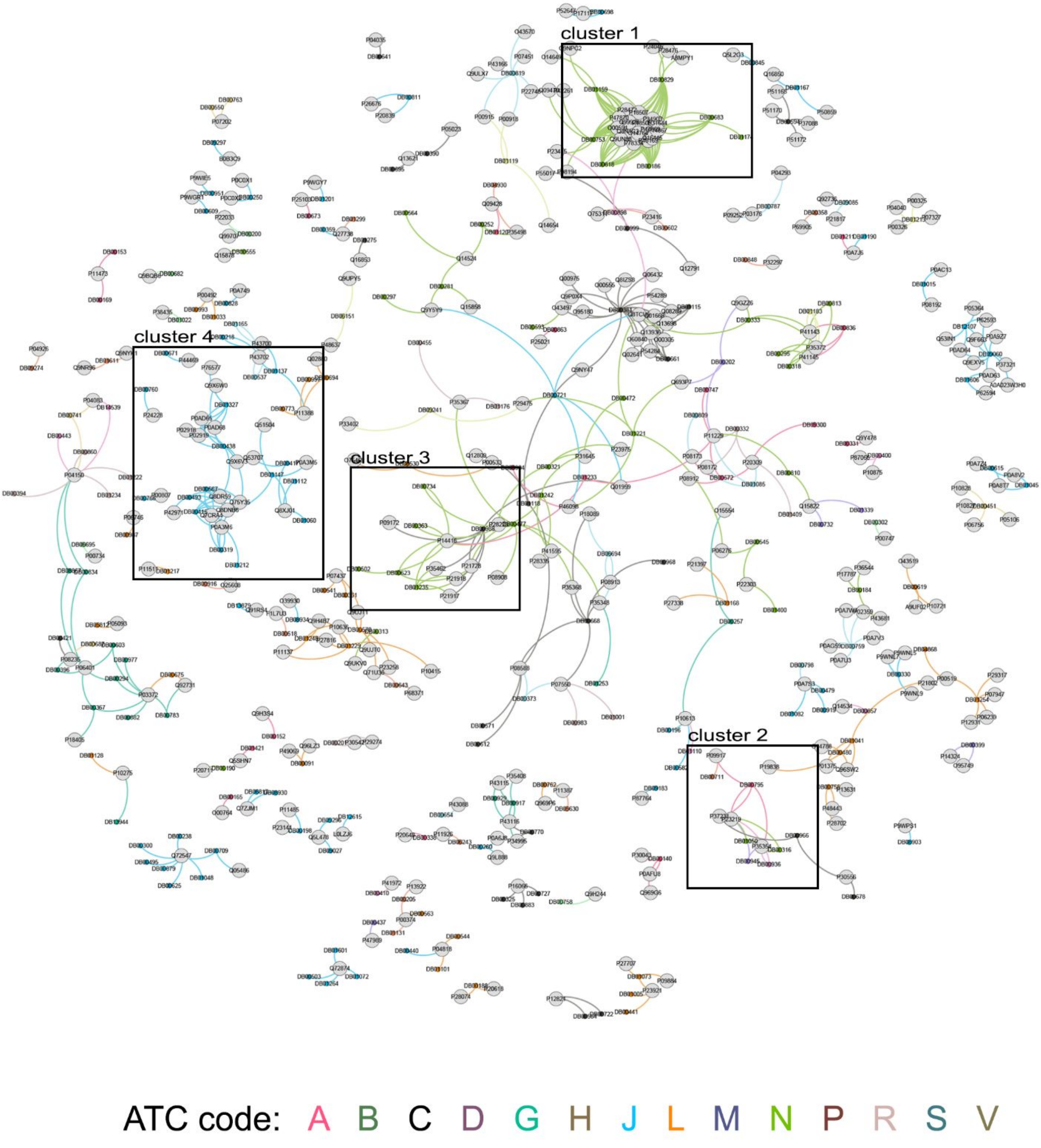
cluster 1
cluster 2
cluster 3
cluster 4
ATC code: A B C D G H J L M N P R S V

Figure S2. The drug–target (DT) network of EMs. The DT network is generated by using the known associations between drugs and targets extracted from the DrugBank database.[1] As of March 2, 2020, DrugBank (version 5.1.5, released 2020-01-03) includes 2635 approved small molecule drugs and 1367 approved biologics. Additionally, 1148 non-redundant proteins (*i.e.* drug target/enzyme/transporter/carrier) sequences are linked to these drug entries. Small and big circles correspond to drugs and target proteins, respectively. The labels correspond to DrugBank and Uniprot codes, respectively. A link is placed between a drug node and a target node if the protein is a known target of that drug. Drug nodes and connecting links are colored according to the ATC code of the drug.

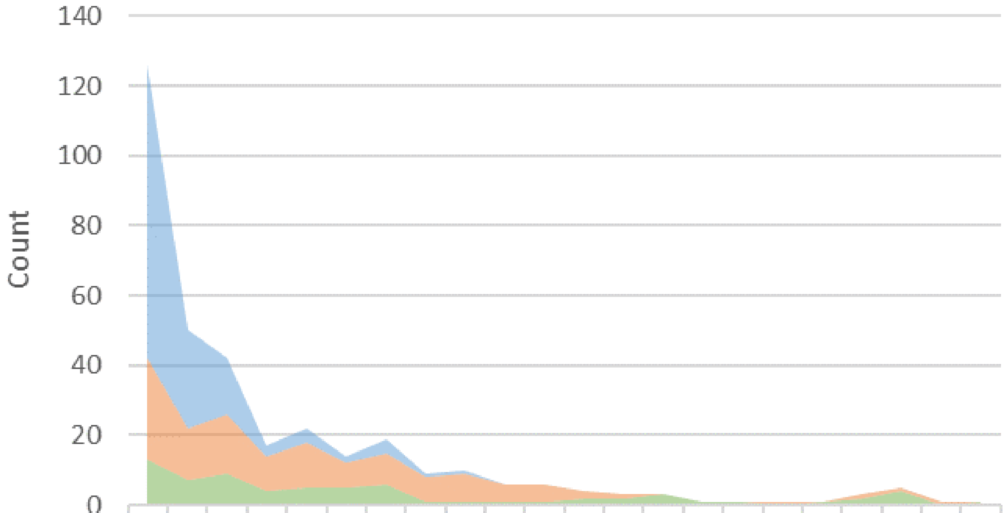

# chiral centers	0	1	2	3	4	5	6	7	8	9	10	11	12	13	14	15	16	17	18	19	20	21
de novo	84	28	16	3	4	2	4	1	1	0	0	0	0	0	0	0	0	0	0	0	0	0
nature-inspired	29	15	17	10	13	7	9	7	8	5	5	2	1	0	0	0	1	0	1	1	1	0
natural	13	7	9	4	5	5	6	1	1	1	1	2	2	3	1	1	0	1	2	4	0	1

Figure S3. Distribution of chiral centers in de novo, Nature-inspired and natural drugs.

Table S1. Properties of cutaneous EMs.

Cutaneous EM	MW, Da	clogP	Melting point, °C
aciclovir	225.2	-0.95	256
benzoyl peroxide	242.23	2.75	103-106
benzyl benzoate	212.24	3.43	21
betamethasone valerate	392.46	3.78	232
fluorouracil	130.08	-0.58	282-283
hydrocortisone acetate	404.5	2.31	220
lidocaine	235.35	1.81	68.5
miconazole	416.13	5.86	170.5
mupirocin	499.61	2.25	77-78
permethrin	391.29	6.24	34
salicylic acid	137.11	1.96	158
terbinafine	292.44	5.51	>212
urea	60.06	-1.80	132.7

Abbreviations: clogP, calculated logP; clogP were extracted using DrugBank[1] or ChemSpider[2] databases.

Table S2. Salient features and classification of EMs reported in the 21st EML.

EM NAME	DRUGBANK ID CODE	CLASSIFICATION			ATC CODE/S	FDA year of approval*
		LEVEL A	LEVEL B	LEVEL C		
abacavir	DB01048	molecular entity	organic	Nature-inspired	J05AR04; J05AR13; J05AF06; J05AR02	1998
abiraterone	DB05812	molecular entity	organic	Nature-inspired	L02BX03	2011
acetazolamide	DB00819	molecular entity	organic	de novo	S01EC01	1953
acetic acid	DB03166	molecular entity	organic	natural	G01AD02; S02AA10	1960
acetylcysteine	DB06151	molecular entity	organic	Nature-inspired	V03AB23; S01XA08; R05CB01	1963
acetylsalicylic acid	DB00945	molecular entity	organic	Nature-inspired	A01AD05; N02BA71; C10BX12; C10BX02; C10BX08; N02BA01; B01AC56; M01BA03; C10BX06; N02BA51; C10BX04; C10BX05; C10BX01; B01AC06	1965
aciclovir	DB00787	molecular entity	organic	Nature-inspired	S01AD03; J05AB01; D06BB53; D06BB03	1982
adalimumab	DB00051	biological entity	proteins		L04AB04	
albendazole	DB00518	molecular entity	organic	de novo	P02CA03	1996
alcohol based hand rub		others	mixtures			
allopurinol	DB00437	molecular entity	organic	Nature-inspired	M04AA51; M04AA01	1966
all-trans retinoid acid	DB00755	molecular entity	organic	natural	L01XX14; D10AD01	1971
alprostadil	DB00770	molecular entity	organic	natural	G04BE01; C01EA01	1981
alteplase	DB00009	biological entity	proteins		B01AD02; S01XA13	
amidotrizoate	DB00271	others	diagnostics		V08AA; V08AA01	
amikacin	DB00479	molecular entity	organic	Nature-inspired	D06AX12; J01RA06; J01GB06; S01AA21	1981
amiloride	DB00594	molecular entity	organic	de novo	C03DB01	1981
amiodarone	DB01118	molecular entity	organic	Nature-inspired	C01BD01	1985
amitriptyline	DB00321	molecular entity	organic	de novo	N06AA09; N06CA01	1961
amlodipine	DB00381	molecular entity	organic	de novo	C09BB03; C09BB07; C10BX03; C10BX07; C09DB07; C08GA02; C09DB01; C09BB04; C09DB05; C09DB02; C09DB06; C09DB04; C09BX01; C09DX03; C09XA53; C10BX11; C09XA54; C09DX01; C08CA01; C09BX03; C10BX14; C10BX09	1992
amodiaquine	DB00613	molecular entity	organic	Nature-inspired	P01BF03; P01BA06	

EM NAME	DRUGBANK ID CODE	CLASSIFICATION			ATC CODE/S	FDA year of approval*
		LEVEL A	LEVEL B	LEVEL C		
amoxicillin	DB01060	molecular entity	organic	Nature-inspired	A02BD07; A02BD01; A02BD06; A02BD04; J01CR02; A02BD11; A02BD05; J01CA04; A02BD10; A02BD03	1974
amphotericin B	DB00681	molecular entity	organic	natural	G01AA03; J02AA01; A07AA07; A01AB04	1966
ampicillin	DB00415	molecular entity	organic	Nature-inspired	J01CR01; S01AA19; J01CA51; J01CA01	1965
anastrozole	DB01217	molecular entity	organic	de novo	L02BG03	1995
anti-D immunoglobulin	DB11597	biological entity	proteins		J06BB01	
Anti-rabies immunoglobulin		biological entity	proteins		J06BB21	
Anti-tetanus immunoglobulin	DB11604	biological entity	proteins		J06BB02	
antivenom immunoglobulin		biological entity	proteins		J06	
aprepitant	DB00673	molecular entity	organic	de novo	A04AD12	2003
arsenic trioxide	DB01169	molecular entity	inorganic		L01XX27	
artemether	DB06697	molecular entity	organic	Nature-inspired	P01BF01; P01BF05; P01BE02	2009
artesunate	DB09274	molecular entity	organic	Nature-inspired	P01BF06; P01BF04; P01BF03; P01BE03; P01BF02	2019
ascorbic acid	DB00126	molecular entity	organic	natural	S01XA15; A11GB01; G01AD03; A11GA01	2017
asparaginase	DB00023	biological entity	proteins		L01XX02	
atazanavir	DB01072	molecular entity	organic	de novo	J05AR15; J05AE08	2003
atracurium	DB00732	molecular entity	organic	Nature-inspired	M03AC04	1983
atropine	DB00572	molecular entity	organic	natural	S01FA01; A03BA01; A03CB03	2014
avibactam	DB09060	molecular entity	organic	de novo		2015
azathioprine	DB00993	molecular entity	organic	Nature-inspired	L04AX01	1968
azithromycin	DB00207	molecular entity	organic	Nature-inspired	S01AA26; J01RA07; J01FA10	1991
barium sulfate	Not Available	others	diagnostics		V08BA02; V08BA01	
BCG vaccine	DB12768	biological entity	proteins		L03AX19	
beclometasone dipropionate	DB00394	molecular entity	organic	Nature-inspired	R03BA01; R03AL09; R01AD01; A07EA07; D07CC04; R03AK08; D07AC15; R03AK13	1976
bedaquiline	DB08903	molecular entity	organic	de novo	J04AK05	2012
bendamustine	DB06769	molecular entity	organic	de novo	L01AA09	2008

EM NAME	DRUGBANK ID CODE	CLASSIFICATION			ATC CODE/S	FDA year of approval*
		LEVEL A	LEVEL B	LEVEL C		
benznidazole	DB11985	molecular entity	organic	de novo	P01CA02	2017
benzoyl peroxide	DB09096	molecular entity	organic	de novo	D10AE51; D10AE01	2018
benzyl benzoate	DB00676	molecular entity	organic	natural	P03AX01	1976
benzylpenicillin	DB01053	molecular entity	organic	natural	J01CE01; J01CE09; S01AA14	1952
betamethasone valerate	DB00443	molecular entity	organic	Nature-inspired	D07AC01; R03BA04; S03BA03; D07XC01; S01CA05; S03CA06; D07BC01; S01BA06; S01CB04; S02BA07; R01AD06; S01BB04; D07CC01; C05AA05; A07EA04; H02AB01	1983
bevacizumab	DB00112	biological entity	proteins		L01XC07	
bicalutamide	DB01128	molecular entity	organic	de novo	L02BB03	1995
biperiden	DB00810	molecular entity	organic	Nature-inspired	N04AA02	1959
bisoprolol	DB00612	molecular entity	organic	Nature-inspired	C09BX02; C07BB07; C07AB07; C07AB57; C07FB07	1992
bleomycin	DB00290	molecular entity	organic	natural	L01DC01	1973
bortezomib	DB00188	molecular entity	organic	de novo	L01XX32	2003
budesonide	DB01222	molecular entity	organic	Nature-inspired	A07EA06; R03AK07; R01AD05; R03BA02; R03AK12; D07AC09	1994
bupivacaine	DB00297	molecular entity	organic	de novo	N01BB01; N01BB51	1972
caffeine	DB00201	molecular entity	organic	natural	V04CG30; N06BC01; R03DA20; N06BC01	1993
calamine		others	mixtures			
calcium		molecular entity	inorganic			
calcium folinate	DB00650	molecular entity	organic	Nature-inspired	V03AF03; V03AF06; V03AF03	2006
calcium gluconate	DB11126	molecular entity	organi	natural	A12AA03; D11AX03	1990
capecitabine	DB01101	molecular entity	organic	Nature-inspired	L01BC06	1998
carbamazepine	DB00564	molecular entity	organic	de novo	N03AF01	1968
carbetocin	DB01282	biological entity	proteins		H01BB03	
carbidopa	DB00190	molecular entity	organic	Nature-inspired	N04BA	1975
carboplatin	DB00958	molecular entity	organic	de novo	L01XA02	1989
cefalexin	DB00567	molecular entity	organic	Nature-inspired	J01DB01	1971
cefazolin	DB01327	molecular entity	organic	Nature-inspired	J01DB04	1973

EM NAME	DRUGBANK ID CODE	CLASSIFICATION			ATC CODE/S	FDA year of approval*
		LEVEL A	LEVEL B	LEVEL C		
cefixime	DB00671	molecular entity	organic	Nature-inspired	J01DD08	1989
cefotaxime	DB00493	molecular entity	organic	Nature-inspired	J01DD01; J01DD51	1981
ceftazidime	DB00438	molecular entity	organic	Nature-inspired	J01DD02; J01DD52	1985
ceftriaxone	DB01212	molecular entity	organic	Nature-inspired	J01DD54; J01DD04	1984
cefuroxime	DB01112	molecular entity	organic	Nature-inspired	J01RA03; S01AA27; J01DC02	1983
charcoal,activated		others	mixtures			
chlorambucil	DB00291	molecular entity	organic	de novo	L01AA02	1957
chloramphenicol	DB00446	molecular entity	organic	natural	D10AF03; S03AA08; D06AX02; J01BA01; S02AA01; G01AA05; S01AA01	1953
chloramphenicol palmitate	DB00394	molecular entity	organic	Nature-inspired	D07CC04; R03AL09; R03BA01; D07AC15; R03AK08; R03AK13; R01AD01; A07EA07	
chloramphenicol succinate	DB07565	molecular entity	organic	Nature-inspired		1959
chlorhexidine	DB00878	molecular entity	organic	de novo	B05CA02; S02AA09; D08AC52; D08AC02; S03AA04; S01AX09; D09AA12; A01AB03; R02AA05	1976
chlorine base compound		others	mixtures			
chloroquine	DB00608	molecular entity	organic	Nature-inspired	P01BA01	1949
chloroxylenol	DB11121	molecular entity	organic	de novo	D08AE05	1980
chlorpromazine	DB00477	molecular entity	organic	de novo	N05AA01	1957
cholera vaccine		biological entity	proteins		J07AE	
ciclosporin	DB00091	molecular entity	organic	natural	L04AD01; S01XA18	1983
ciprofloxacin	DB00537	molecular entity	organic	de novo	S01AE03; S03AA07; J01RA12; J01MA02; J01RA10; S02AA15; J01RA11	1987
cisplatin	DB00515	molecular entity	inorganic		L01XA01	
clarithromycin	DB01211	molecular entity	organic	Nature-inspired	A02BD07; A02BD06; A02BD04; A02BD09; J01FA09; A02BD05	1991
clavulanic acid	DB00766	molecular entity	organic	natural	J01CR	1984
clindamycin	DB01190	molecular entity	organic	Nature-inspired	J01FF01; D10AF01; D10AF51; G01AA10	1970
clindamycin palmitate	DB01190	molecular entity	organic	Nature-inspired	J01FF01; D10AF01; D10AF51; G01AA10	1986
clofazimine	DB00845	molecular entity	organic	de novo	J04BA01	1986
clomifene	DB00882	molecular entity	organic	de novo	G03GB02	1967

EM NAME	DRUGBANK ID CODE	CLASSIFICATION			ATC CODE/S	FDA year of approval*
		LEVEL A	LEVEL B	LEVEL C		
clomipramine	DB01242	molecular entity	organic	de novo	N06AA04	1989
clopidogrel	DB00758	molecular entity	organic	de novo	B01AC04	1997
clotrimazole	DB00257	molecular entity	organic	de novo	G01AF02; D01AC01; A01AB18	1975
cloxacillin	DB01147	molecular entity	organic	Nature-inspired	J01CF02	1971
clozapine	DB00363	molecular entity	organic	de novo	N05AH02	1989
Coagulation Factor IX Human	DB13152	biological entity	proteins		B02BD04	
Coagulation Factor VIII Human	DB14473	biological entity	proteins		B02BD14	
coal tar		others	mixtures			
codeine	DB00318	molecular entity	organic	natural	R05DA04; N02AA59; N02AA79	1950
colecalciferol	DB00169	molecular entity	organic	natural	M05BB07; M05BB05; M05BB04; M05BB08; A11CC05; M05BX53; M05BB03	
colistin	DB00803	molecular entity	organic	natural	J01XB01; A07AA10	2007
condoms		others	devices			
copper-containing device		others	devices			
cyclizine	DB01176	molecular entity	organic	de novo	R06AE03; R06AE53	1966
cyclophosphamide	DB00531	molecular entity	organic	de novo	L01AA01	1959
cycloserine	DB00260	molecular entity	organic	natural	J04AB01	1964
cytarabine	DB00987	molecular entity	organic	Nature-inspired	L01BC01	1969
dabigatran etexilate	DB06695	molecular entity	organic	de novo	B01AE07	2010
dacarbazine	DB00851	molecular entity	organic	de novo	L01AX04	1975
daclatasvir	DB09102	molecular entity	organic	de novo	J05AX14	2015
dactinomycin	DB00970	molecular entity	organic	natural	L01DA01	1964
dapsone	DB00250	molecular entity	organic	de novo	D10AX05; J04BA02	1979
darunavir	DB01264	molecular entity	organic	de novo	J05AR14; J05AE10	2006
dasabuvir	DB09183	molecular entity	organic	de novo	J05AX16; J05AX66	2014
dasatinib	DB01254	molecular entity	organic	de novo	L01XE06	2006
daunorubicin	DB00694	molecular entity	organic	natural	L01DB02	1979

EM NAME	DRUGBANK ID CODE	CLASSIFICATION			ATC CODE/S	FDA year of approval*
		LEVEL A	LEVEL B	LEVEL C		
deferoxamine	DB00746	molecular entity	organic	natural	V03AC01	1968
delamanid	DB11637	molecular entity	organic	de novo	J04AK06	
dengue vaccine	DB14764	biological entity	proteins		J07BX	
desmopressin	DB00035	biological entity	proteins		H01BA02	
dexamethasone phosphate	DB01234	molecular entity	organic	Nature-inspired	D07XB05; S01CA01; S03CA01; D07CB04; A01AC02; S03BA01; H02AB02; S01BA01; R01AD53; S01CB01; D10AA03; S02BA06; S02CA06; D07AB19; C05AA09; R01AD03	1959
dextran 70	DB09255	others	mixtures			
diaphragms		others	devices			
diazepam	DB00829	molecular entity	organic	de novo	N05BA01	1963
diazoxide	DB01119	molecular entity	organic	de novo	C02DA01; V03AH01	1973
diethylcarbamazine	DB00711	molecular entity	organic	de novo	P02CB02	1982
digoxin	DB00390	molecular entity	organic	natural	C01AA05	1954
dihydroartemisinin	DB11638	molecular entity	organic	Nature-inspired	P01BF05; P01BF; P01BE05	
diloxanide furoate	DB14638	molecular entity	organic	de novo		
dimercaprol	DB06782	molecular entity	organic	de novo	V03AB09	1946
dinoprostone	DB00917	molecular entity	organic	natural		1977
diphtheria antitoxin		biological entity	proteins		J06AA01	
diphtheria vaccine		biological entity	proteins		J07AF	
docetaxel	DB01248	molecular entity	organic	Nature-inspired	L01CD02	1996
docusate sodium	DB11089	molecular entity	organic	de novo	A06AA02; A06AG10	1900
dolutegravir	DB08930	molecular entity	organic	de novo	J05AX12; J05AR13	2013
dopamine	DB00988	molecular entity	organic	natural	C01CA04	1974
doxorubicin	DB00997	molecular entity	organic	natural	L01DB01	1974
doxycycline	DB00254	molecular entity	organic	Nature-inspired	A01AB22; J01AA02	1967
efavirenz	DB00625	molecular entity	organic	de novo	J05AG03; J05AR11; J05AR06	1998
eflornithine	DB06243	molecular entity	organic	Nature-inspired	P01CX03; D11AX16	2000

EM NAME	DRUGBANK ID CODE	CLASSIFICATION			ATC CODE/S	FDA year of approval*
		LEVEL A	LEVEL B	LEVEL C		
emtricitabine	DB00879	molecular entity	organic	Nature-inspired	J05AR17; J05AR19; J05AR09; J05AR08; J05AR18; J05AR06; J05AR03; J05AF09	2003
enalapril	DB00584	molecular entity	organic	Nature-inspired	C09BB02; C09AA02; C09BB06; C09BA02	1985
enoxaparin	DB01225	biological entity	carbohydrates		B01AB05	
entecavir	DB00442	molecular entity	organic	Nature-inspired	J05AF10	2005
ephedrine	DB01364	molecular entity	organic	natural	S01FB02; R01AB05; R01AA03; A08AA56; R03CA02; C01CA26	1948
epinephrine	DB00668	molecular entity	organic	natural	R03AA01; B02BC09; A01AD01; R01AA14; C01CA24; S01EA51; R03AK01; S01EA01	1951
ergocalciferol	DB00153	molecular entity	organic	natural	A11CC01	1941
ergometrine	DB01253	molecular entity	organic	natural	G02AB03	2006
erlotinib	DB00530	molecular entity	organic	de novo	L01XE03	2004
erythromycin	DB00199	molecular entity	organic	natural	J01FA01; S01AA17; D10AF52; D10AF02	1964
erythropoiesis-stimulating agents		biological entity	proteins		B03XA	
estradiol cypionate	DB00783	molecular entity	organic	Nature-inspired	G03CA03	1979
ethambutol	DB00330	molecular entity	organic	de novo	J04AM06; J04AK02; J04AM03	1967
ethanol	DB00898	molecular entity	organic	natural	D08AX08; V03AB16; V03AZ01	1990
ethinylestradiol	DB00977	molecular entity	organic	Nature-inspired	G03AA16; G03AA03; G03AA08; L02AA03; G03AA13; G03CA01	1973
ethionamide	DB00609	molecular entity	organic	de novo	J04AD03	1965
ethosuximide	DB00593	molecular entity	organic	de novo	N03AD51; N03AD01	1960
etonogestrel	DB00294	molecular entity	organic	Nature-inspired	G03AC08	2001
etoposide	DB00773	molecular entity	organic	Nature-inspired	L01CB01	1983
fentanyl	DB00813	molecular entity	organic	Nature-inspired	N02AB03; N01AH01; N01AH51	1968
ferrous salt		molecular entity	inorganic			
fexinidazole	DB12265	molecular entity	organic	de novo	P01CA03	
filgrastim	DB00099	biological entity	proteins		L03AA02	
fluconazole	DB00196	molecular entity	organic	de novo	D01AC15; J02AC01; J01RA07	1990
flucytosine	DB01099	molecular entity	organic	Nature-inspired	D01AE21; J02AX01	1971

EM NAME	DRUGBANK ID CODE	CLASSIFICATION			ATC CODE/S	FDA year of approval*
		LEVEL A	LEVEL B	LEVEL C		
fludarabine	DB01073	molecular entity	organic	Nature-inspired	L01BB05	1991
fludrocortisone acetate	DB00687	molecular entity	organic	Nature-inspired	S01CA06; H02AA02; H02AA02; S02CA07; S03CA05	1955
fluorescein	DB00693	others	diagnostics		S01JA01; S01JA51	
fluorouracil	DB00544	molecular entity	organic	Nature-inspired	L01BC02; L01BC52	1962
fluoxetine	DB00472	molecular entity	organic	de novo	N06CA03; N06AB03	1987
fluphenazine decanoate	DB00623	molecular entity	organic	de novo	N05AB02	1967
fluphenazine enantate	DB00623	molecular entity	organic	de novo	N05AB02	1987
folic acid	DB00158	molecular entity	organic	natural	B03AE01; B03BB01; B03BB51	1947
fomepizole	DB01213	molecular entity	organic	de novo	V03AB34	1997
formoterol	DB00983	molecular entity	organic	Nature-inspired	R03AK07; R03AK11; R03AK09; R03AK08; R03AC13; R03AL05	2001
fosfomycin	DB00828	molecular entity	organic	natural	J01XX01	1996
fresh frozen plasma		others	mixtures			
furosemide	DB00695	molecular entity	organic	de novo	C03CB01; C03CA01; C03EB01	1966
gemcitabine	DB00441	molecular entity	organic	Nature-inspired	L01BC05	1996
gentamicin	DB00798	molecular entity	organic	natural	S01AA11; S03AA06; D06AX07; J01GB03; S02AA14	1973
glecaprevir	DB13879	molecular entity	organic	de novo	J05AP57	2017
gliclazide	DB01120	molecular entity	organic	de novo	A10BB09	
glucagon	DB00040	biological entity	proteins		H04AA01	
glucose	DB01914	molecular entity	organic	natural	C05BB56; V06DC01; B05CX01; V04CA02	1953
glutaral	DB03266	molecular entity	organic	de novo	D08AX09	
griseofulvin	DB00400	molecular entity	organic	natural	D01AA08; D01BA01	1962
Haemophilus influenzae type b vaccine		biological entity	proteins		J07AG01	
haloperidol	DB00502	molecular entity	organic	de novo	N05AD01	1967
halothane	DB01159	molecular entity	organic	de novo	N01AB01	1958
heparin sodium	DB01109	biological entity	carbohydrates		C05BA; S01XA14; C05BA53; S01XA; B01AB51; B01AB; B01AB01; C05BA03	
hepatitis A vaccine		biological entity	proteins		J07BC02	

EM NAME	DRUGBANK ID CODE	CLASSIFICATION			ATC CODE/S	FDA year of approval*
		LEVEL A	LEVEL B	LEVEL C		
hepatitis B vaccine		biological entity	proteins		J07BC01	
HPV vaccine		biological entity	proteins		J07BM03	
hydralazine	DB01275	molecular entity	organic	de novo	C02DB02; C02LG02	1953
hydrochlorothiazide	DB00999	molecular entity	organic	de novo	C09DX03; C09XA54; C03AX01; C03AA03; C09DX01; C03AB03; C09XA52; C09BX03; C03EA01	1959
hydrocortisone	DB00741	molecular entity	organic	natural	S02BA01; A07EA02; C05AA01; A01AC03; S01CB03; R01AD60; S01BA02; D07AA02; D07XA01; S01BB01; S02CA03; D07BA04; H02AB09; S03CA04; D07CA01; S01CA03	1952
hydrocortisone acetate	DB14539	molecular entity	organic	Nature-inspired		1954
hydrocortisone succinate	DB14545	molecular entity	organic	Nature-inspired		1955
hydroxocobalamin	DB00200	molecular entity	organic	natural	B03BA03; V03AB33; B03BA53	1975
hydroxycarbamide	DB01005	molecular entity	organic	natural	L01XX05	1998
hydroxychloroquine	DB01611	molecular entity	organic	Nature-inspired	P01BA02	1955
hyoscine butylbromide	DB09300	molecular entity	organic	Nature-inspired	A03BB01; A03DB04	1979
hyoscine hydrobromide	DB00747	molecular entity	organic	natural	A04AD51; A04AD01; S01FA02; N05CM05	1979
ibuprofen	DB01050	molecular entity	organic	de novo	C01EB16; M02AA13; M01AE51; N02AJ19; R02AX02; N02AJ08; G02CC01; M01AE01	1974
ifosfamide	DB01181	molecular entity	organic	de novo	L01AA06	1987
imatinib	DB00619	molecular entity	organic	de novo	L01XE01	2001
influenza vaccine		biological entity	proteins		J07BB02	
insulin injection	DB00030	biological entity	proteins		A10AC01; A10AD01; A10AE01; A10A; A10AF01; A10AB01	
intermediate-acting insulin		biological entity	proteins		A10AC	
intraperitoneal dialysis solution		others	solutions			
iodine	DB05382	molecular entity	inorganic		D08AG03	
iohexol	DB01362	others	diagnostics		V08AB02	
ipratropium	DB00332	molecular entity	organic	Nature-inspired	R01AX03; R03AL01; R03AL02; R03BB01	1986
irinotecan	DB00762	molecular entity	organic	Nature-inspired	L01XX19	1996

EM NAME	DRUGBANK ID CODE	CLASSIFICATION			ATC CODE/S	FDA year of approval*
		LEVEL A	LEVEL B	LEVEL C		
isoflurane	DB00753	molecular entity	organic	de novo	N01AB06	1979
isoniazid	DB00951	molecular entity	organic	Nature-inspired	J04AM02; J04AC01; J04AM03; J04AM04; J04AM06; J04AC51; J04AM01; J04AM05	1952
isosorbide dinitrate	DB00883	molecular entity	organic	Nature-inspired	C01DA08; C01DA58; C05AE02	1959
itraconazole	DB01167	molecular entity	organic	de novo	J02AC02	1992
ivermectin	DB00602	molecular entity	organic	Nature-inspired	P02CF01; D11AX22	1996
Japanese encephalitis vaccine		biological entity	proteins		J07BA	
ketamine	DB01221	molecular entity	organic	de novo	N01AX03	1970
lactic acid	DB04398	molecular entity	organic	natural	G01AD01; QP53AG02	1971
lactulose	DB00581	molecular entity	organic	Nature-inspired	A06AD11; A06AD61	1976
lamivudine	DB00709	molecular entity	organic	Nature-inspired	J05AF05; J05AR11; J05AR05; J05AR04; J05AR02; J05AR01; J05AR27; J05AR12; J05AR16; J05AR13; J05AR07	1995
lamotrigine	DB00555	molecular entity	organic	de novo	N03AX09	1994
latanoprost	DB00654	molecular entity	organic	Nature-inspired	S01EE01	1996
ledipasvir	DB09027	molecular entity	organic	de novo	J05AX65	2014
lenalidomide	DB00480	molecular entity	organic	de novo	L04AX04	2005
leuprorelin	DB00007	biological entity	proteins		L02AE02	
levamisole	DB00848	molecular entity	organic	de novo	P02CE01	1990
levodopa	DB01235	molecular entity	organic	natural	N04BA03; N04BA01; N04BA02	1970
levofloxacin	DB01137	molecular entity	organic	de novo	J01MA12; A02BD10; J01RA05; S01AE05	1996
levonorgestrel	DB00367	molecular entity	organic	Nature-inspired	G03FA11; G03FB09; G03FB01; G03AD01; G03AC03; G03AB03; G03FA10; G03AA07; G03AA06	1990
levothyroxine	DB00451	molecular entity	organic	natural	H03AA01	2000
lidocaine	DB00281	molecular entity	organic	de novo	D04AB01; C05AD01; S01HA07; R02AD02; N01BB02; S02DA01; C01BB01; N01BB52	1948
linezolid	DB00601	molecular entity	organic	de novo	J01XX08	2000
lisinopril	DB00722	molecular entity	organic	Nature-inspired	C09BA03; C09BB03; C10BX07; C09AA03	1987
lithium carbonate	DB01356	molecular entity	inorganic		N05AN01	

EM NAME	DRUGBANK ID CODE	CLASSIFICATION			ATC CODE/S	FDA year of approval*
		LEVEL A	LEVEL B	LEVEL C		
loperamide	DB00836	molecular entity	organic	Nature-inspired	A07DA03; A07DA53	1976
lopinavir	DB01601	molecular entity	organic	de novo	J05AR10	2000
loratadine	DB00455	molecular entity	organic	de novo	R06AX13	1993
lorazepam	DB00186	molecular entity	organic	de novo	N05BA56; N05BA06	1977
losartan	DB00678	molecular entity	organic	de novo	C09CA01; C09DA01; C09DB06	1995
Lugol's solution	DB14492	other	solutions			
lumefantrine	DB06708	molecular entity	organic	de novo	P01BF01	2009
magnesium sulfate	DB00653	molecular entity	inorganic		A12CC02; D11AX05; B05XA05; V04CC02; A06AD04	
mannitol	DB00742	molecular entity	organic	natural	R05CB16; B05BC01; B05CX04; A06AD16	1964
measles vaccine		biological entity	proteins		J07BD	
mebendazole	DB00643	molecular entity	organic	de novo	P02CA51; P02CA01	1974
medroxyprogesterone acetate	DB00603	molecular entity	organic	Nature-inspired	G03AC06; G03DA02; L02AB02	1959
mefloquine	DB00358	molecular entity	organic	Nature-inspired	P01BC02; P01BF02	1989
meglumine antimoniate	DB13732	molecular entity	organic	Nature-inspired	P01CB01; QP51AB01	
meglumine iotroxate	DB08945	others	diagnostics		V08AC02	
melarsoprol	DB12864	molecular entity	organic	de novo	P01CD01	
melphalan	DB01042	molecular entity	organic	de novo	L01AA03	1964
meningococcal meningitis		biological entity	proteins		J07AH	
mercaptopurine	DB01033	molecular entity	organic	Nature-inspired	L01BB02	1953
meropenem	DB00760	molecular entity	organic	Nature-inspired	J01DH02; J01DH52	1996
mesna	DB09110	molecular entity	organic	de novo	V03AF01; R05CB05	1988
metformin	DB00331	molecular entity	organic	Nature-inspired	A10BD05; A10BD03; A10BD11; A10BD07; A10BD17; A10BD20; A10BD18; A10BD08; A10BD10; A10BD13; A10BD14; A10BA02; A10BD16; A10BD15; A10BD02	1995
methadone	DB00333	molecular entity	organic	Nature-inspired	N02AC52; N07BC02	1947
methimazole	DB00763	molecular entity	organic	de novo	H03BB52; H03BB02	1950

EM NAME	DRUGBANK ID CODE	CLASSIFICATION			ATC CODE/S	FDA year of approval*
		LEVEL A	LEVEL B	LEVEL C		
methotrexate	DB00563	molecular entity	organic	Nature-inspired	L04AX03; L01BA01	1953
methyldopa	DB00968	molecular entity	organic	Nature-inspired	C02LB01; C02AB01	1962
methylprednisolone succinate	DB14644	molecular entity	organic	Nature-inspired	H02BX01; D10AA02; D07CA02; S01CA08; D07AA01; H02AB04	1959
methylthioninium chloride	DB09241	molecular entity	organic	de novo	V04CG05; V03AB17	1990
metoclopramide	DB01233	molecular entity	organic	de novo	A03FA01	1979
metronidazole	DB00916	molecular entity	organic	de novo	A01AB17; D06BX01; A02BD01; J01RA03; A02BD11; A02BD08; P01AB51; G01AF01; J01XD01; J01RA10; P01AB01; A02BD02; J01RA04; A02BD03	1963
metronidazole benzoate		molecular entity	organic	de novo	A01AB17; D06BX01; A02BD01; J01RA03; A02BD11; A02BD08; P01AB51; G01AF01; J01XD01; J01RA10; P01AB01; A02BD02; J01RA04; A02BD03	1963
miconazole	DB01110	molecular entity	organic	de novo	J02AB01; A07AC01; G01AF04; D01AC02; A01AB09; D01AC52; S02AA13	1978
midazolam	DB00683	molecular entity	organic	de novo	N05CD08	1985
mifepristone	DB00834	molecular entity	organic	Nature-inspired	G03XB51; G03XB01	2000
miltefosine	DB09031	molecular entity	organic	Nature-inspired	L01XX09	2014
misoprostol	DB00929	molecular entity	organic	Nature-inspired	M01AE56; G02AD06; A02BB01	1988
morphine	DB00295	molecular entity	organic	natural	N02AA01; N02AG01; N02AA51; A07DA52; R05DA05	1971
moxifloxacin	DB00218	molecular entity	organic	de novo	J01MA14; S01AE07	1999
multiple micronutrient		others	mixtures			
mumps vaccine		biological entity	proteins		J07BE01	
mupirocin	DB00410	molecular entity	organic	natural	R01AX06; D06AX09	1987
naloxone	DB01183	molecular entity	organic	Nature-inspired	N02AA53; A06AH04; V03AB15	1971
natamycin	DB00826	molecular entity	organic	natural	S01AA10; A07AA03; D01AA02; G01AA02; A01AB10	1978
neostigmine	DB01400	molecular entity	organic	Nature-inspired	N07AA51; S01EB06; N07AA01	1973
nevirapine	DB00238	molecular entity	organic	de novo	J05AR05; J05AR07; J05AG01	1996
niclosamide	DB06803	molecular entity	organic	de novo	P02DA01	1982
nicotinamide	DB02701	molecular entity	organic	natural	A11HA01	1988

EM NAME	DRUGBANK ID CODE	CLASSIFICATION			ATC CODE/S	FDA year of approval*
		LEVEL A	LEVEL B	LEVEL C		
nicotine replacement therapy	DB00184	molecular entity	organic	natural	N07BA01	1984
nifedipine	DB01115	molecular entity	organic	de novo	C08CA05; C08GA01; C08CA55	1981
nifurtimox	DB11820	molecular entity	organic	de novo	P01CC01	
nilotinib	DB04868	molecular entity	organic	de novo	L01XE08	2007
nitrofurantoin	DB00698	molecular entity	organic	de novo	J01XE01; J01XE51	1953
nitroglycerin	DB00727	molecular entity	organic	Nature-inspired	C01DA02; C05AE01; C01DA52	1981
nitrous oxide	DB06690	molecular entity	inorganic		N01AX63; N01AX13	
nivolumab	DB09035	biological entity	proteins		L01XC17	
norethisterone	DB00717	molecular entity	organic	Nature-inspired	G03AA05; G03AC01; G03FB05; G03DC02; G03FA01; G03AB04	1973
norethisterone enantate	DB14678	molecular entity	organic	Nature-inspired	G03DC02	1973
normal immunoglobulin		biological entity	proteins		J06BA	
nystatin	DB00646	molecular entity	organic	natural	D01AA01; G01AA51; A07AA02; G01AA01	1964
ofloxacin	DB01165	molecular entity	organic	de novo	S01AE01; J01MA01; S02AA16; J01RA09	1990
ombitasvir	DB09296	molecular entity	organic	de novo	J05AX67; J05AX66	2015
omeprazole	DB00338	molecular entity	organic	de novo	A02BC01; A02BD05; A02BD01	1989
ondansetron	DB00904	molecular entity	organic	de novo	A04AA01	1991
oral rehydration		others	solutions			
oral rehydration salts		others	solutions			
oseltamivir	DB00198	molecular entity	organic	Nature-inspired	J05AH02	1999
oxaliplatin	DB00526	molecular entity	organic	de novo	L01XA03	2002
oxamniquine	DB01096	molecular entity	organic	de novo	P02BA02	1980
oxygen	DB09140	molecular entity	inorganic		V03AN01	
oxytocin	DB00107	biological entity	proteins		G02AC01; H01BB02	
paclitaxel	DB01229	molecular entity	organic	natural	L01CD01; L01CD03	1992
p-aminosalicylic acid	DB00233	molecular entity	organic	Nature-inspired	J04AA03; J04AA01; J04AA02	1994
pancreatic enzymes		biological entity	proteins			
paracetamol	DB00316	molecular entity	organic	de novo	N02BE01; N02BE51; N02BE71	1968

EM NAME	DRUGBANK ID CODE	CLASSIFICATION			ATC CODE/S	FDA year of approval*
		LEVEL A	LEVEL B	LEVEL C		
paritaprevir	DB09297	molecular entity	organic	de novo	J05AX67; J05AX66	2014
paromomycin	DB01421	molecular entity	organic	natural	A07AA06	1969
pegaspargase	DB00059	biological entity	proteins		L01XX24	
pegylated interferon alfa 2a		biological entity	proteins		L03AB15	
penicillamine	DB00859	molecular entity	organic	Nature-inspired	M01CC01	1970
pentamidine	DB00738	molecular entity	organic	de novo	P01CX01	1984
permethrin	DB04930	molecular entity	organic	Nature-inspired	P03AC54; P03AC04	1986
pertussis vaccine		biological entity	proteins		J07AJ	
phenobarbital	DB01174	molecular entity	organic	de novo	N03AA02	1966
phenoxymethylpenicillin	DB00417	molecular entity	organic	natural	J01CE02	1995
phenytoin	DB00252	molecular entity	organic	de novo	N03AB02; N03AB52	1953
phytomenadione	DB01022	molecular entity	organic	natural	B02BA01	1955
pibrentasvir	DB13878	molecular entity	organic	de novo	J05AP57	2017
pilocarpine	DB01085	molecular entity	organic	natural	S01EB01; N07AX01; S01EB51	1974
piperacillin	DB00319	molecular entity	organic	Nature-inspired	J01CR05; J01CA12	1981
piperaquine	DB13941	molecular entity	organic	de novo	P01BF05; P01BX02	
platelets		others	blood and derivatives			
plazomicin	DB12615	molecular entity	organic	Nature-inspired	J01GB14	2018
pneumococcal vaccine		biological entity	proteins		J07AL	
podophyllum resin	DB09094	others	mixtures			
poliomyelitis vaccine		biological entity	proteins		J07BF	
polymyxin B	DB00781	molecular entity	organic	natural	S01AA18; J01XB02; S02AA11; S03AA03; A07AA05	1964
potassium chloride	DB00761	molecular entity	inorganic		B05XA01; A12BA51; A12BA01	
potassium ferric hexacyano-ferrate(II - 2H20		molecular entity	inorganic			
potassium iodide	DB06715	molecular entity	inorganic		R05CA02; V03AB21; S01XA04	
potassium permanganate	DB13831	molecular entity	inorganic		D08AX06; V03AB18	

EM NAME	DRUGBANK ID CODE	CLASSIFICATION			ATC CODE/S	FDA year of approval*
		LEVEL A	LEVEL B	LEVEL C		
povidone iodine	DB06812	others	solutions		R02AA15; D09AA09; G01AX11; D08AG02; S01AX18; D11AC06	
praziquantel	DB01058	molecular entity	organic	de novo	P02BA01	1982
prednisolone	DB00860	molecular entity	organic	Nature-inspired	D07AA03; R01AD02; D07XA02; S01BB02; S02BA03; D07BA01; S03CA02; S01CB02; C05AA04; S01CA02; H02AB06; A01AC54; R01AD52; D07CA03; S02CA01; V03AB05; S03BA02; S01BA04; A07EA01	1955
primaquine	DB01087	molecular entity	organic	de novo	P01BA03	1952
procaine	DB00721	molecular entity	organic	de novo	J01CE09	1948
procarbazine	DB01168	molecular entity	organic	de novo	L01XB01	1969
progesterone	DB00396	molecular entity	organic	natural	G03DA04; G03FA04	1976
proguanil	DB01131	molecular entity	organic	Nature-inspired	P01BB51; P01BB01	1948
propofol	DB00818	molecular entity	organic	de novo	N01AX10	1989
propranolol	DB00571	molecular entity	organic	Nature-inspired	C07AA05; C07FA05; C07BA05	1967
propylthiouracil	DB00550	molecular entity	organic	Nature-inspired	H03BA02	1947
protamine sulfate	DB09141	biological entity	proteins		V03AB14	
pyrantel	DB11156	molecular entity	organic	de novo	P02CC01	2001
pyrazinamide	DB00339	molecular entity	organic	Nature-inspired	J04AK01; J04AM06; J04AM05	1971
pyridostigmine	DB00545	molecular entity	organic	Nature-inspired	N07AA02	1955
pyridoxine	DB00165	molecular entity	organic	natural	A11HA02	1972
pyrimethamine	DB00205	molecular entity	organic	de novo	P01BD51; P01BD01; P01BF04	1953
pyronaridine	DB12975	molecular entity	organic	de novo	P01BF06	
quinine	DB00468	molecular entity	organic	natural	M09AA72; P01BC01	2005
rabies vaccine		biological entity	proteins		J07BG01	
raltegravir	DB06817	molecular entity	organic	de novo	J05AR16; J05AX08	2007
ranitidine	DB00863	molecular entity	organic	de novo	A02BA02	1983
realgar-Indigo naturalis		others	mixtures			
red blood cells		others	blood and derivatives			

EM NAME	DRUGBANK ID CODE	CLASSIFICATION			ATC CODE/S	FDA year of approval*
		LEVEL A	LEVEL B	LEVEL C		
retinol palmitate		molecular entity	organic	Nature-inspired		1949
ribavirin	DB00811	molecular entity	organic	Nature-inspired	J05AB04	1985
riboflavin	DB00140	molecular entity	organic	natural	A11HA04	1970
rifabutin	DB00615	molecular entity	organic	Nature-inspired	J04AB04	1992
rifampicin	DB01045	molecular entity	organic	Nature-inspired	J04AB02	1971
rifapentine	DB01201	molecular entity	organic	Nature-inspired	J04AB05	1998
risperidone	DB00734	molecular entity	organic	de novo	N05AX08	1993
ritonavir	DB00503	molecular entity	organic	de novo	J05AX67; J05AX66; J05AR10; J05AE03	1996
rituximab	DB00073	biological entity	proteins		L01XC02	
rotavirus vaccine		biological entity	proteins		J07BH	
rubella vaccine		biological entity	proteins		J07BJ	
salbutamol	DB01001	molecular entity	organic	Nature-inspired	R03AC02; R03CC02; R03AK04; R03AL02; R03AK13	1981
salicylic acid	DB00936	molecular entity	organic	natural	N02BA16; N02BA12; D01AE12; N02BA08; S01BC08; N02BA03; N02BA04	1938
selenium sulfide	DB00971	molecular entity	inorganic		D01AE13	
senna		others	mixtures			
simvastatin	DB00641	molecular entity	organic	Nature-inspired	C10AA01; C10BA02; C10BA04; C10BX04; C10BX01	1991
sodium calcium edetate	DB14598	molecular entity	organic	de novo		2009
sodium chloride	DB09153	molecular entity	inorganic		B05XA03; B05CB01; S01XA03; A12CA01	
sodium fluoride	DB09325	molecular entity	inorganic		A01AA51; A01AA01; A12CD01	
sodium hydrogen carbonate		molecular entity	inorganic			
sodium nitrite	DB09112	molecular entity	inorganic		V03AB08	
sodium nitroprusside	DB00325	molecular entity	organic	Nature-inspired		1974
sodium stibogluconate	DB05630	molecular entity	organic	Nature-inspired	P01CB02	
sodium thiosulfate	DB09499	molecular entity	inorganic			
sofosbuvir	DB08934	molecular entity	organic	Nature-inspired	J05AX69; J05AX65; J05AX15	2013
spectinomycin	DB00919	molecular entity	organic	natural	J01XX04	1971
spironolactone	DB00421	molecular entity	organic	Nature-inspired	C03DA01	1960

EM NAME	DRUGBANK ID CODE	CLASSIFICATION			ATC CODE/S	FDA year of approval*
		LEVEL A	LEVEL B	LEVEL C		
streptokinase	DB00086	biological entity	proteins		B01AD01; B06AA55	
streptomycin	DB01082	molecular entity	organic	natural	A07AA04; J01GA01; A07AA54; J04AM01	1946
succimer	DB00566	molecular entity	organic	de novo	V03AB	1991
sulfadiazine	DB00359	molecular entity	organic	de novo	J01EE06; J01EC02; J01EE02	1941
sulfadoxine	DB01299	molecular entity	organic	Nature-inspired	QJ01EW13	1981
sulfamethoxazole	DB01015	molecular entity	organic	Nature-inspired	J01EE01; J01EC01	1973
sulfasalazine	DB00795	molecular entity	organic	Nature-inspired	A07EC01	1950
suramin	DB04786	molecular entity	organic	de novo	P01CX02; P01CX02	
surfactant		others	mixtures			
suxamethonium	DB00202	molecular entity	organic	Nature-inspired	M03AB01	1952
tamoxifen	DB00675	molecular entity	organic	de novo	L02BA01	1977
tazobactam	DB01606	molecular entity	organic	Nature-inspired	J01CG02	1993
telmisartan	DB00966	molecular entity	organic	de novo	C09DB04; C09CA07; C09DA07	1998
tenofovir disoproxil	DB00300	molecular entity	organic	Nature-inspired	J05AR11; J05AR09; J05AR24; J05AR08; J05AF07; J05AR06; J05AR12; J05AR03; J05AF07	2001
terbinafine	DB00857	molecular entity	organic	de novo	D01AE15; D01BA02	1992
testosterone enanthate	DB13944	molecular entity	organic	Nature-inspired	G03EA02; G03BA03	1953
tetanus vaccine		biological entity	proteins		J07AM	
tetracaine	DB09085	molecular entity	organic	de novo	S01HA03; N01BA03; C05AD02; N01BA53; D04AB06	1958
tetracycline	DB00759	molecular entity	organic	natural	J01AA07; D06AA04; J01RA08; A02BD08; S01AA09; S03AA02; A01AB13; A02BD02; S02AA08	1953
thalidomide	DB01041	molecular entity	organic	de novo	L04AX02	1998
thiamine	DB00152	molecular entity	organic	natural	A11DA01	1972
tick-borne encephalitis vaccine		biological entity	proteins		J07BA	
timolol	DB00373	molecular entity	organic	Nature-inspired	S01ED51; S01ED01; C07BA06; C07DA06; C07AA06	1981
tioguanine	DB00352	molecular entity	organic	Nature-inspired	L01BB03	1966
tiotropium	DB01409	molecular entity	organic	Nature-inspired	R03AL06; R03BB04; R03BB54	2004
tranexamic acid	DB00302	molecular entity	organic	Nature-inspired	B02AA02	1986

EM NAME	DRUGBANK ID CODE	CLASSIFICATION			ATC CODE/S	FDA year of approval*
		LEVEL A	LEVEL B	LEVEL C		
trastuzumab	DB00072	biological entity	proteins		L01XC03	
triclabendazole	DB12245	molecular entity	organic	de novo	P02BX04	2019
trimethoprim	DB00440	molecular entity	organic	Nature-inspired	J01EA01; J01EE03; J01EE07; J01EE04; J01EE01; J01EE02; J01EE05	1980
tropicamide	DB00809	molecular entity	organic	Nature-inspired	S01FA56; S01FA06	1960
tuberculin,purified protein derivative	DB11601	others	diagnostics			
typhoid vaccine		biological entity	proteins		J07AP	
ulipristal acetate	DB08867	molecular entity	organic	Nature-inspired	G03XB02; G03AD02	2010
urea	DB03904	molecular entity	organic	natural	B05BC02; D02AE01; D02AE51	1995
vaborbactam	DB12107	molecular entity	organic	de novo	J01DH52	2017
valganciclovir	DB01610	molecular entity	organic	Nature-inspired	J05AB14	2001
valproic acid	DB00313	molecular entity	organic	de novo	N03AG01	1978
vancomycin	DB00512	molecular entity	organic	natural	A07AA09; J01XA01	1964
varicella vaccine		biological entity	proteins		J07BK	
vecuronium	DB01339	molecular entity	organic	Nature-inspired	M03AC03	1984
velpatasvir	DB11613	molecular entity	organic	de novo	J05AX69	2016
verapamil	DB00661	molecular entity	organic	de novo	C08DA51; C08DA01; C09BB10	1981
vinblastine	DB00570	molecular entity	organic	natural	L01CA01	1965
vincristine	DB00541	molecular entity	organic	natural	L01CA02	1963
vinorelbine	DB00361	molecular entity	organic	Nature-inspired	L01CA04	1994
voriconazole	DB00582	molecular entity	organic	de novo	J02AC03	2002
warfarin	DB00682	molecular entity	organic	natural	B01AA03	1954
water for injection		others	solutions			
whole blood		others	blood and derivatives			
xylometazoline	DB06694	molecular entity	organic	de novo	S01GA53; R01AA07; R01AB06; S01GA03	1959
yellow fever vaccine		biological entity	proteins		J07BL01	
zidovudine	DB00495	molecular entity	organic	Nature-inspired	J05AR04; J05AF01; J05AR01; J05AR05	1987

EM NAME	DRUGBANK ID CODE	CLASSIFICATION			ATC CODE/S	FDA year of approval*
		LEVEL A	LEVEL B	LEVEL C		
zinc sulfate	DB09322	molecular entity	inorganic		A12CB01	
zoledronic acid	DB00399	molecular entity	organic	de novo	M05BA08; M05BB08	2001

* extracted (when available) for organic drugs only.

References:

1. Wishart, D. S.; Knox, C.; Guo, A. C.; Shrivastava, S.; Hassanali, M.; Stothard, P.; Chang, Z.; Woolsey, J. Drugbank: a comprehensive resource for in silico drug discovery and exploration. *Nucleic Acids Res.* **2006**, *34*, D668-D672.
2. http://www.chemspider.com/ (accessed Mar 5 , 2020).
